# Occurrence, Severity and Temporal Trends of Enzootic Pneumonia-like Lung Lesions in Hungarian Slaughter Pigs, 2018–2022: Insights from Zero-Inflated Mixed-Effects Models

**DOI:** 10.3390/ani16193033

**Published:** 2026-09-26

**Authors:** Bettina Paszerbovics, Péter Máté, Andrea Harnos, László Ózsvári

**Affiliations:** 1 Department of Biostatistics, University of Veterinary Medicine Budapest, 1078 Budapest, Hungary; harnos.andrea@univet.hu; 2 Veterinary Practitioner, H-7630 Pecs, Hungary; matepvet@gmail.com; 3 Centre for Translational Medicine, Semmelweis University, 1085 Budapest, Hungary; 4 Department of Veterinary Forensics and Economics, University of Veterinary Medicine Budapest, 1078 Budapest, Hungary; ozsvari.laszlo@univet.hu

**Keywords:** enzootic pneumonia, slaughterhouse monitoring, lung lesions, Madec score, swine health, zero-inflated model

## Abstract

Porcine respiratory disease complex remains a challenge for pig production, and slaughterhouse lung inspection provides a tool for respiratory health monitoring. This study investigated the occurrence, severity, temporal trends, and anatomical distribution of enzootic pneumonia-like lung lesions in Hungarian slaughter pigs between 2018 and 2022 and evaluated associations with farm size. Of the 13,535 lungs examined, 13,499 with complete lobe-level data were analysed using the Madec scoring system, based on 174 slaughterhouse examinations across 38 farms. Zero-inflated mixed-effects models with a negative binomial distribution were used to analyse lesion occurrence and lesion scores. EP-like lesions (Madec score ≥ 1) occurred in 25.3% of lungs, declining from 57.4% in 2018 to 8.6% in 2022, and mean Madec scores decreased from 2.7 to 0.3. Most lungs (91.0%) scored between 0 and 4, whereas 9.0% had moderate-to-severe lesions (scores 5–28). Lesions were most frequent and severe in the cardiac and apical lobes and showed greater involvement of the right-sided lobes. Farm size was not significantly associated with either model component, although structural zero probabilities were lowest for small and highest for large farms. Structural-zero probability increased over time, indicating that declining overall Madec scores mainly reflected structural zeros rather than conditional-score changes.

## Simple Summary

Respiratory diseases are health problems in pigs, reducing animal welfare and farm profitability. Information on lung health in pigs from Central and Eastern Europe remains limited compared with Western Europe. Examination of lungs at slaughter provides a practical way to monitor respiratory health. In this study, lung lesions typical of enzootic pneumonia were assessed in slaughter pigs from Hungarian farms between 2018 and 2022. The study also investigated the relationship between farm size and lung health, as well as which lung regions were most affected by lesions. An advanced statistical approach enabled a more accurate analysis of the data than conventional methods. The proportion of pigs with lung lesions decreased from more than half of the pigs examined in 2018 to less than one tenth in 2022. Lesions were most frequent and severe in the cardiac and apical lobes and occurred more often in the right lung. The observed proportion of pigs with detectable lung lesions was higher in small farms than in medium-sized and large farms; however, the statistical models did not provide evidence of a significant farm-size effect. These findings support slaughterhouse monitoring as a useful tool for describing respiratory-health patterns in examined pig populations.

## 1. Introduction

The porcine respiratory disease complex (PRDC) remains a formidable challenge for the global pig industry. This complex condition arises from the interplay of viral and bacterial infections, coupled with environmental, housing, and management factors [1,2]. PRDC has significant economic impact in pig farming. Manifestations include reduced growth performance and feed efficiency, prolonged fattening periods, increased antibiotic use, and economic losses due to elevated mortality. The severity and consequent financial losses depend on the specific pathogens involved, as well as housing and management factors [3,4]. For monitoring subclinical PRDC, abattoir lung inspection is an optimal method for assessing lesion occurrence and severity [5], providing effective and valuable feedback to farm owners on herd health status [6]. Abattoir monitoring utilizes established scoring systems to assess lung conditions and pleuritis in pigs, employing well-established and standardized methods [7,8,9]. Primary lung findings identified during abattoir inspection include enzootic pneumonia (EP)-like lesions and pleuritis [5,6]. Microorganisms like *Mycoplasma hyopneumoniae*, *Pasteurella multocida*, *Actinobacillus pleuropneumoniae*, swine influenza viruses, porcine circovirus type 2 (PCV2) and porcine reproductive and respiratory syndrome (PRRS) virus are the main causative agents [10,11,12]. Published data on the prevalence of EP-like lesions in slaughter pigs are scarce in Hungary. Only limited slaughterhouse-based lung health data are available from Central-Eastern Europe, highlighting the need for updated epidemiological information on respiratory lesions in commercial pig populations.

Previous European studies have demonstrated that lung lesion distribution is not uniform, with higher lesion frequency and severity reported in specific lung lobes and on the right side of the lung, reflecting anatomical asymmetry. These observations support the separate evaluation of lung sides and individual lobes when assessing EP-like lesions at slaughter [13,14,15]. In addition to infectious agents, several investigations have emphasised the significant contribution of non-infectious factors to lung health [16,17], among which farm size has been identified as a possible factor influencing lesion prevalence and severity [18].

Slaughterhouse lung-scoring data are typically characterised by a high proportion of zero values. These zeros may reflect either an absence of detectable lesions at the herd level or the absence of detectable lesions in individual animals despite ongoing infection within the herd. Conventional analytical approaches that do not distinguish between these mechanisms may produce biased estimates of disease occurrence and severity. Zero-inflated models provide a suitable framework to address this challenge by explicitly separating structural from sampling zeros [19].

The aims of this study were: (i) to describe the temporal trends, overall occurrence, and lobe-specific distribution of EP-like lung lesions in slaughter pigs in Hungary between 2018 and 2022 using the Madec scoring system; (ii) to evaluate the association between farm size and the occurrence and severity of EP-like lung lesions; and (iii) to apply statistical modelling approaches that appropriately account for zero inflation and repeated observations in slaughterhouse lung-scoring data.

## 2. Materials and Methods

### 2.1. Herds and Data Collection

A total of 174 slaughterhouse examinations were conducted between 2018 and 2022, comprising 38 distinct farms from six Hungarian regions. Data were obtained from Southern Hungary (19 farms; 5909 pigs), Western Hungary (4 farms; 4610 pigs), Central-Western Hungary (7 farms; 2100 pigs), Central Hungary (1 farm; 430 pigs), Northeastern Hungary (4 farms; 333 pigs), and Southeastern Hungary (3 farms; 153 pigs). Convenience sampling was applied. Farms and slaughterhouses were included if they met the following criteria: (1) an annual slaughter pig production of at least 5000 pigs per farm; (2) availability of complete farm production and health records during the study period; (3) willingness of farm management to participate in the study and permit data collection at slaughter; and (4) permission granted by the slaughterhouse for on-site access.

In each slaughterhouse investigation, the sample size ranged from 20 to 100 slaughter pigs. The 38 distinct farms included in the study were classified into three farm size categories based on their annual number of finishing pigs. Farms with <15,000 slaughter pigs per year were classified as small (15 farms), those with 15,000–50,000 slaughter pigs as medium (13 farms), and farms with >50,000 slaughter pigs as large (10 farms). This categorisation was defined to reflect the professional and production-level considerations relevant to Hungarian pig farming. The examined farms represented a range of production systems, including fully integrated farrow-to-finish operations, segregated multi-site production systems, and finishing-only units. The herds consisted predominantly of pigs originating from the Topigs, DanBred, Hypor and PIC genetic lines.

### 2.2. Slaughterhouse and Lung Inspections

The lungs and pleura of slaughter pigs were examined by visual inspection and palpation. The assessment involved scoring pigs’ lungs using the Madec scoring system. Enzootic pneumonia-like lesions were evaluated per lobe as follows: 0 = no lesions; 1 = lesions affecting ≤ 25% of the lobe; 2 = lesions affecting 26–50% of the lobe; 3 = lesions affecting 51–75% of the lobe; and 4 = lesions affecting > 75% of the lobe [7]. Examination results were initially recorded on paper forms or audio records and subsequently transferred to Microsoft Excel (Microsoft Corp., Redmond, WA, USA) spreadsheets for data analysis.

For each examination, information regarding the company, herd, number of slaughtered and examined animals, date of examination, attending veterinarian, and examining veterinarian was recorded. Where available, information on the age of the animals at slaughter, the weight of the animals, the room and/or stable of origin was also collected.

Assessments were conducted in a blinded manner, meaning that the examiner had no prior knowledge of the identity or management characteristics of the herd being evaluated. No lung samples were collected for microbiological or molecular confirmation; therefore, the observed lesions were classified as EP-like lesions based on macroscopic appearance and Madec scoring, rather than confirmed etiologically.

Written consent for the analysis and publication of anonymised and aggregated data was obtained.

### 2.3. Data Analysis

For the analysis of lung lesions, Madec lung lobe scores were evaluated both at the individual lobe level and as a summed score. For lobe-specific investigations, Madec scores were analysed separately for each lung lobe. In addition, a Madec sum score was calculated for each pig by summing the scores of the seven lung lobes, according to the Madec scoring system [7]. This yielded a single composite score per animal representing the overall lung lesion burden. Both summed and lobe-specific Madec scores were averaged by year for descriptive purposes and subsequently used as outcome variables in the statistical models. In the modelling framework, Madec scores were treated as count data because the scoring system quantifies the extent of affected lung tissue by counting affected lobe quarters. Consequently, higher scores reflect a greater extent of diseased lung tissue, supporting the use of count-based analytical approaches.

### 2.4. Statistical Analysis

Statistical analyses were designed to account for the hierarchical structure of the data and the high proportion of zero scores. Repeated observations were present at multiple levels, including farms, slaughterhouse examinations (batches) and individual animals. EP-like lung lesions were analysed using zero-inflated generalised mixed-effects models [20]. For the analysis of overall lung lesion burden, the Madec sum score across seven lung lobes was used as the dependent variable. Year and farm size were included as categorical fixed effects, together with their interaction, with farm and slaughterhouse examination (batch) as nested random effects (batch nested within farm). The zero-inflation component included additive effects of year and farm size, with farm and slaughterhouse examination (batch) as nested random effects (batch nested within farm). The distribution of Madec scores was characterised by a large excess of zero values, substantially exceeding the number expected under conventional count distributions. Conceptually, excess zeros may arise from heterogeneity in the underlying lesion-generating process. Zero-inflated models allow these excess zeros to be represented by a latent zero-inflation component, while the count component accounts for observations arising from the lesion-score generating process. Observed zero scores may arise under different underlying conditions, including the absence of detectable lesions in animals with differing levels of pathogen exposure. In the zero-inflated model, the structural-zero component is latent and therefore cannot be directly linked to a specific biological mechanism or infection status. The conditional count component may also generate zero scores. Conventional count models assume that all zeros arise from a single stochastic process and therefore may not adequately represent the underlying biological structure. To account for these distinct sources of zero observations, while simultaneously modelling lesion burden in the conditional count component, zero-inflated generalised mixed-effects models with a negative binomial distribution were applied. To investigate whether lesion severity and occurrence differed within lung regions, lobes were first combined into four lobe categories: apical (left and right apical lobes), cardiac (left and right cardiac lobes), diaphragmatic (left and right diaphragmatic lobes), and accessory. To evaluate potential left–right asymmetry, the original seven-lobe classification was further collapsed into two categories: left (left apical, left cardiac, and left diaphragmatic lobes) and right (right apical, right cardiac, and right diaphragmatic lobes). Data from the accessory lobe were excluded from this analysis.

For analyses of lobe-specific lesion scores among lobe categories (apical, cardiac, diaphragmatic, and accessory) or lung sides (left and right), individual lobe-specific Madec scores were used as outcomes. For the lobe-region analysis, lobe region, year, and farm size were included as fixed effects, together with the lobe region and year interaction in the conditional component. For the left–right analysis, lung side, year, and farm size were included as additive fixed effects in the conditional component. In both models, farm and animal identity nested within farm were included as random effects to account for clustering of observations within farms and repeated measurements of multiple lung lobes within the same animal. The zero-inflation component included year, anatomical region (lobe region or lung side), and their interaction, with the same nested random-effects structure as the conditional component. The models were fitted using a negative binomial distribution family.

Model diagnostics were assessed using simulation-based residual diagnostics implemented in DHARMa. For each retained zero-inflated negative-binomial model, simulation-based residual diagnostics were generated using 1000 simulations. Diagnostic assessment included graphical inspection of simulated residuals, residual patterns by year and farm, and formal assessment of zero inflation. The distribution of the estimated random effects was additionally evaluated using quantile–quantile (QQ) plots. Because formal tests can be sensitive to small deviations in large datasets, diagnostic test results were interpreted together with graphical assessments rather than in isolation.

To assess whether the observed temporal pattern could be influenced by changes in the composition of sampled farms across years, a sensitivity analysis was performed restricted to repeatedly sampled farms, defined as farms represented in at least two distinct study years. The Madec sum score model was refitted in this restricted dataset using the same model specification as in the primary analysis. Because the Madec sum score is theoretically bounded between 0 and 28, whereas the negative-binomial distribution has no fixed upper bound, an additional simulation-based assessment was performed to evaluate the upper-tail behaviour of the fitted model. A total of 1000 datasets were simulated from the final fitted model. The proportion of simulated Madec sum scores exceeding the theoretical maximum of 28 was calculated, together with the 95th, 97.5th, and 99th percentiles of the pooled simulated response distribution. As a sensitivity analysis of farm-size contrasts, a farm-level cluster bootstrap with 500 attempted resamples was performed, with farms resampled with replacement as complete clusters. Of the original 500 attempted resamples, 403 produced usable model fits. Increasing the number of attempted resamples to 665 yielded 541 usable model fits; 123 unsuccessful fits had a non-positive-definite Hessian, and one failed because of an NA/NaN (Not Available/ Not a Number) gradient evaluation. Percentile 95% confidence intervals were obtained for farm-size contrasts in the conditional and zero-inflation components, with intervals excluding 1 considered evidence of a farm-size difference.

As sensitivity analyses, alternative models were fitted using different representations of the Madec score. These models were not considered assumption-free reference models or replacements for the primary zero-inflated negative-binomial model but were used to assess whether the main qualitative conclusions were sensitive to the distributional assumptions and outcome representation of the primary analysis. An ordinal mixed-effects model was fitted as a sensitivity analysis, treating the Madec sum score as an ordered outcome. This analysis preserves the ordering and bounded nature of the score but relies on assumptions specific to the ordinal model and addresses a different estimand from the primary count-based model. Madec sum scores were categorised into three ordered severity categories (0, 1–4, and 5–28) and analysed using a cumulative link mixed-effects model with a logit link. These categories provided interpretable severity levels while accounting for the highly skewed distribution; using all possible sum scores would have resulted in sparse upper categories and a substantially more complex model. A binomial mixed-effects model was additionally fitted after dichotomising the Madec sum score as zero (score = 0) versus non-zero (score > 0). Although dichotomisation discards information on lesion extent, this analysis provided a direct sensitivity assessment of temporal and farm-size patterns in the probability of a non-zero Madec score without relying on the latent zero-inflation classification. Both models included year, farm size, and their interaction as fixed effects, with random intercepts for farm and batch nested within farm. The small-farm and 2022 combination was excluded because no lung lesions were observed in this group, preventing reliable model estimation.

Corresponding simulation-based assessments were performed for the lobe-region and lung-side models, for which individual lobe scores are theoretically bounded between 0 and 4. For each model, 1000 datasets were simulated and the frequency of simulated values exceeding 4, together with selected upper quantiles of the simulated response distribution, was evaluated. For the lobe-region and lung-side sensitivity analyses, ordinal models were fitted to individual lobe scores using their original five ordered categories (0, 1, 2, 3, and 4). These models were fitted to the lobe-region dataset (94,493 lobe-level observations) and the left–right dataset excluding the accessory lobe (80,994 observations). However, reliable estimation was not achieved within a reasonable computational time, including after model simplification, and the ordinal models were therefore not used for inference. Binomial models were also fitted after dichotomising individual lobe scores as lesion absent (score = 0) or present (score > 0). The lobe-region model included lobe region, year, farm size, and the lobe region and year interaction as fixed effects, whereas the lung-side model included lung side, year, farm size, and the lung side and year interaction. Both models included random intercepts for farm and animal identity nested within farm.

For predictions from the zero-inflated negative-binomial models, overall expected scores incorporated both model components and were calculated as E(Y)=μ∗1−π, where *E*(*Y*) is the overall expected score, μ is the conditional mean and π is the estimated zero-inflation probability [21]. Model-based point estimates were obtained on the overall response scale with random effects set to zero. Thus, these estimates represent predictions conditional on zero-valued random effects rather than marginal means integrated over the estimated random-effects distributions. Because uncertainty in these overall predictions depends jointly on the conditional and zero-inflation components, 95% confidence intervals were obtained by simulation from the joint asymptotic multivariate normal distribution of the conditional and zero-inflation fixed-effect coefficients, using their full estimated covariance matrix. A total of 100,000 coefficient draws were generated for each analysis, and the 2.5th and 97.5th percentiles of the resulting overall expected responses were used as the confidence limits. For the Madec sum-score analysis, year-specific overall predictions were additionally averaged with equal weights across farm-size categories to characterise the overall temporal pattern. For the lobe-region and lung-side analyses, predictions were similarly averaged with equal weights across farm-size categories when farm size was not the factor of interest. All analyses were performed using R version 4.3.2 (R Foundation for Statistical Computing, Vienna, Austria). Zero-inflated mixed-effects models were fitted using the glmmTMB function from the glmmTMB package (version 1.1.13) [20] and cumulative link mixed-effects models were fitted using the clmm function from the ordinal package (version 2025.12.29) [22]. Estimated overall means and pairwise comparisons were obtained using functions from the emmeans package (version 2.0.1) [23], including emmeans, regrid, and pairs; omnibus effects of categorical predictors were evaluated using joint Wald χ^2^ tests of estimable contrasts implemented with the joint_tests function. Simulation-based confidence intervals for overall response-scale predictions were generated using multivariate normal draws from the estimated joint fixed-effect covariance matrix with the mvrnorm function from the MASS package (version 7.3.61) [24]. Simulated residuals were generated with the simulateResiduals function from the DHARMa package (version 0.4.7) [25]. Statistical significance was evaluated at the 0.05 level (*p* < 0.05) and pairwise comparisons were adjusted for multiple testing using Tukey’s method. The complete R script used for all statistical analyses is provided as Supplementary File.

## 3. Results

Between 2018 and 2022, a total of 174 slaughterhouse examinations were conducted, resulting in the macroscopic assessment of 13,535 pig lungs from 38 farms (Table 1). Although 38 unique farms were included, repeated investigations yielded 66 farm-year observations over the study period.

### 3.1. Occurrence and Severity of EP-like Lesions

Overall, EP-like lesions were detected in approximately one-quarter of the examined lungs. Of the 13,535 lungs examined at the slaughterhouse, 36 were excluded from further analysis because one or more lung lobes were missing and the Madec sum score could therefore not be calculated. Consequently, the Madec sum score was available for 13,499 lungs, which were included in the descriptive and model-based analyses. EP-like lesions (Madec score ≥ 1) were identified in 25.3% (3419/13,499) of lungs. Scores ranged from 0 to 28, with a median of 0 and a mean of 1.1. The distribution of Madec scores was strongly right-skewed. Most lungs (91.0%) had scores between 0 and 4, indicating the absence or mild lesions: score 0 in 74.7% (10,080/13,499); score 1 in 6.4% (859/13,499); score 2 in 4.3% (576/13,499); score 3 in 2.9% (395/13,499); and score 4 in 2.8% (383/13,499). The remaining 9.0% exhibited moderate-to-severe lesions (scores 5–28).

### 3.2. Temporal Trends

A consistent decline in both the proportion of affected lungs and mean Madec sum scores was observed over the study period. In 2018, EP-like lesions were present in 57.4% of lungs, with a mean Madec score of 2.7. By 2022, lesion occurrence had decreased to 8.6%, and the mean score to 0.3. Intermediate years showed a gradual reduction, indicating a decline in lesion burden across the sampled examinations (Table 2).

### 3.3. Distribution of Lesions Among Lung Lobes

The distribution of EP-like lesions differed among lung lobes. Cardiac lobes consistently showed the highest proportion of non-zero Madec scores, followed by apical lobes, whereas diaphragmatic lobes and the accessory lobe were least affected (Figure 1). This anatomical pattern remained stable across the study period despite the overall decline in lesion frequency.

In addition to score distributions, mean Madec scores were calculated by lobe and year to summarise overall lesion burden (Figure 2). Mean values consistently highlighted cardiac and apical lobes as the most severely affected lobes in each year, whereas diaphragmatic and accessory lobes exhibited substantially lower mean scores throughout the study period. Together, the distributional and mean-based perspectives demonstrate both the distribution of lesion severity within each lobe and the variation in average lesion burden among lobes over time. The distribution of Madec scores across individual lung lobes and years is presented in Supplementary Figure S1.

### 3.4. Analysis of Madec Scores

Overall predicted Madec sum scores, incorporating both the conditional and zero-inflation components of the model and averaged across farm-size categories, declined over the study period. Farm-size-specific predictions showed a clear temporal decline in large farms, whereas estimates for medium and small farms fluctuated among years (model specifications, model-selection criteria, and diagnostic results are provided in Supplementary Tables S1–S3 and Supplementary Figures S2–S5). In the conditional component, Madec sum scores varied among years and farm-size categories, but there was no evidence of a year and farm-size interaction (*p* = 0.2297), and the model did not indicate a consistent temporal decline in conditional lesion scores across farm-size categories. In contrast, the zero-inflation component showed a clear temporal pattern, with the estimated probability of structural zeros increasing markedly overall across all farm-size categories, although a slight decline was observed in 2021 compared with 2020 in each farm-size category. Across years, small farms generally had the lowest estimated probabilities of structural zeros and large farms the highest. However, this descriptive ordering did not constitute evidence of a farm-size effect: only year was significantly associated with the probability of structural zeros (*p* < 0.0001), whereas farm size was not significant (*p* = 0.2112), and no significant pairwise differences were detected among farm-size categories. Thus, the modelled temporal decline in overall Madec scores was primarily associated with the increasing probability of structural zeros rather than a consistent decline in the conditional lesion-score component.

Raw and model-based mean Madec sum scores and estimated probabilities of structural zeros by year and farm-size category are presented in Table 3. Omnibus Wald χ^2^ tests, fixed-effect estimates, random-effect variance estimates, and model-fit statistics for the zero-inflated mixed-effects model are provided in Supplementary Tables S4–S9. Overall model-based predicted Madec sum scores, conditional predicted Madec sum scores, and estimated structural zero probabilities by year and farm-size category are presented in Supplementary Tables S10–S13. Pairwise comparisons of structural zero probabilities among farm-size categories, including odds ratios, 95% confidence intervals (CIs), and adjusted *p*-values, are provided in Supplementary Table S14.

Raw means, overall model-based predictions, conditional-component predictions, and 95% confidence intervals are shown in Figure 3.

Because the farms included in the study varied across calendar years, a sensitivity analysis was conducted using only repeatedly sampled farms. Of the 38 farms, 23 were represented in one study year, 7 in two years, 4 in three years, 3 in four years, and 1 in all five years. Thus, 15 farms were represented in at least two study years and were included in the sensitivity analysis, comprising 11,227 lung examinations. The number of these farms represented in individual years was 5, 8, 10, 12, and 8 in 2018–2022, respectively. Overall predicted Madec sum scores showed a clear temporal decline in large farms but fluctuated in medium and small farms. Conditional mean scores also varied among years, whereas the estimated probability of structural zeros showed an overall increase from 2018 to 2022 across farm-size categories, although a slight decrease was observed between 2020 and 2021 in each category. Thus, restricting the analysis to repeatedly sampled farms did not materially alter the main patterns observed in the full dataset. Detailed results of the sensitivity analysis are presented in Supplementary Tables S15–S18.

An additional simulation-based assessment was used to evaluate the upper-tail behaviour of the fitted model. In the simulated data, 1.49% of Madec sum scores exceeded the theoretical maximum of 28, and the 95th, 97.5th, and 99th percentiles were 12, 20, and 36, respectively. These findings indicate that the fitted model had a heavier upper tail than permitted by the scoring scale, representing a limitation when interpreting predictions at the extreme upper end of the response distribution. The ordinal and binomial analyses showed similar temporal patterns, with a shift towards lower Madec score categories and lower estimated probabilities of having a non-zero Madec score over time, respectively, while these analyses and the farm-level bootstrap provided no consistent evidence of farm-size differences; detailed results are provided in Supplementary Tables S19–S24. Comparisons of three alternative model specifications, including omnibus fixed-effect tests and information criteria, are provided in Supplementary Tables S25–S26.

### 3.5. Lobe- and Side-Specific Patterns of Lung Lesions

The cardiac lobe showed the highest predicted overall mean Madec scores, followed by the apical lobe, whereas the accessory and diaphragmatic lobes generally showed lower and similar scores. In 2018, the cardiac lobe differed significantly from the other regions (all *p* ≤ 0.0080), and the apical lobe also differed significantly from the accessory and diaphragmatic lobes (*p* = 0.0073 and *p* = 0.0030, respectively). From 2019 onwards, pairwise differences among lobes were no longer statistically significant, although the general pattern persisted. Figure 4 illustrates the temporal evolution of predicted overall mean Madec scores for apical, cardiac, diaphragmatic, and accessory lung lobes. The model specification, model-selection criteria and diagnostic plots for the anatomical lobe region analyses are provided in Supplementary Tables S27–S29 and Supplementary Figures S6–S9. Omnibus Wald χ^2^ tests, fixed-effect estimates, predicted overall means and pairwise comparisons of predicted overall means among anatomical lobe regions are presented in Supplementary Tables S30–S34.

The zero-inflation component indicated differences among lung lobes in the probability of belonging to the structural zero class. Across the study period, the cardiac lobe consistently exhibited the lowest estimated structural zero probability, followed by the apical lobe. In all years except 2018 and 2021, the apical lobe had significantly higher odds of belonging to the structural zero class than the cardiac lobe (OR: 1.48–2.14, *p* ≤ 0.0289). In contrast, the diaphragmatic and accessory lobes generally exhibited the highest structural zero probabilities, consistent with lower overall lesion involvement in these lobes. Estimated structural zero probabilities by anatomical lobe region and year are provided in Supplementary Table S35. Complete pairwise comparisons of the odds of structural zeros among anatomical lobe regions within each year, including all odds ratios, 95% confidence intervals (CI), and adjusted *p*-values, are presented in Supplementary Table S36.

Simulation-based assessment was used to evaluate the upper-tail behaviour of the fitted model. In the simulated data, 0.50% of Madec lobe scores exceeded the theoretical maximum of 4, and the 95th, 97.5th, and 99th percentiles were 2, 3, and 4, respectively. These findings indicate that the fitted model occasionally generated values above the theoretical scoring limit, representing a minor limitation when interpreting predictions at the extreme upper end of the response distribution. Further sensitivity analysis treating lobe-level lesion presence across anatomical lobe regions as a binary outcome showed a similar anatomical pattern, with the cardiac lobes generally having higher estimated probabilities of a non-zero Madec score, followed by the apical lobes, whereas the accessory and diaphragmatic lobes generally had lower probabilities. Estimated probabilities also generally decreased over the study period, particularly in the cardiac and apical lobes; detailed results are provided in Supplementary Tables S37–S39.

The right lung generally exhibited higher predicted overall mean Madec scores than the left lung across the study period, although the difference was statistically significant only in 2018 (*p* = 0.0006). However, the estimated difference was small (0.06 Madec score units), suggesting limited biological relevance despite statistical significance. From 2019 onwards, differences between the left and right lung were even smaller and no longer statistically significant (all *p* ≥ 0.1238). The model specifications, model-selection criteria and diagnostic plots for the left-versus-right lung side analyses are provided in Supplementary Tables S40–S42 and Supplementary Figures S10–S13. Omnibus Wald χ^2^ tests, fixed-effect estimates, overall predicted scores and pairwise comparisons of predicted overall mean Madec scores between the left and right lung sides are presented in Supplementary Tables S43–S47.

The zero-inflation component also revealed differences between lung sides. From 2018 to 2021, the left lung had significantly higher odds of belonging to the structural zero class than the right lung (OR: 1.19–1.46, all *p* ≤ 0.0149 depending on the year). However, no significant difference between lung sides was detected in 2022 (OR = 1.08, *p* = 0.4567). Estimated probabilities of structural zeros for the left and right lung sides by year are provided in Supplementary Table S48. Complete pairwise comparisons of the odds of structural zeros between the left and right lung sides, including all odds ratios, 95% confidence intervals (CIs), and adjusted *p*-values, are presented in Supplementary Table S49.

Simulation-based assessment indicated that 0.39% of simulated lobe scores exceeded the theoretical maximum of 4 and the 95th, 97.5th, and 99th percentiles were 2, 3, and 4, respectively. These findings indicate that the fitted model occasionally generated values above the theoretical scoring limit, representing a minor limitation when interpreting predictions at the extreme upper end of the response distribution. Further sensitivity analysis dichotomising lobe-level Madec scores as zero versus non-zero showed a similar right-sided predominance, with higher estimated probabilities of a non-zero Madec score on the right than on the left, although the difference diminished over time and was not statistically significant in 2021 or 2022. The estimated probability of a non-zero Madec score declined markedly on both sides over the study period; detailed results are provided in Supplementary Tables S50–S52.

## 4. Discussion

### 4.1. Temporal Trends in EP-like Lung Lesions

A pronounced decline in enzootic pneumonia (EP)-like lung lesions was observed between 2018 and 2022. Descriptively, the occurrence of EP-like lesions decreased from over 50% to below 10%, while the observed mean Madec sum score declined from 2.7 to 0.3. This temporal pattern was also supported by the model-based estimates, with the predicted overall mean Madec sum score, averaged over farm-size categories, decreasing from 2.7 in 2018 to 0.2 in 2022. Importantly, a sensitivity analysis restricted to the 15 farms represented in at least two study years showed a similar pattern, with the overall mean Madec sum score decreasing from 1.9 in 2018 to 0.3 in 2021; the corresponding estimate for 2022 could not be reliably estimated. The dataset represents one of the most recent multi-regional abattoir-based assessments of porcine lung health in Hungary.

Across Europe, reported prevalences of EP-like lesions at slaughter vary widely, ranging from below 20% to over 90% [26,27]. This variation may be attributable to differences in study design (whether investigations were conducted at national level or limited to selected farms), herd type (e.g., farrow-to-finish farms, finishing farms), geographic scope, diagnostic criteria and methods, time period and sample size [28,29,30,31]. In addition, differences in herd health status, biosecurity measures, vaccination practices, and production systems may contribute to the observed variability [3,32,33,34]. Several European studies have reported EP-like lesions affecting approximately one-third to more than one-half of slaughter pigs, while prevalences exceeding 70–90% have been reported in some intensive production systems [13,27,29,35,36,37,38,39].

Recent data on EP-like lung lesions in Hungarian slaughter pigs are scarce, as lung scoring surveys have rarely been published. In one of the few available studies, conducted in 1997–1998, pneumonia lesions were detected in around 80% of pigs [40]. The findings of the present study indicate a marked improvement in lung health within the investigated Hungarian pig population. The low proportion of pigs with EP-like lesions and low mean lung scores observed in the later years of the study place this population within the lower range of contemporary European observations.

The observed decline in EP-like lung lesions is likely multifactorial. Enzootic pneumonia-like lesions are influenced by several respiratory pathogens and management-related factors [15,34]. Among these, porcine reproductive and respiratory syndrome virus (PRRSV) is considered a major contributor to respiratory disease complexes, as it impairs pulmonary immune defences and increases susceptibility to secondary bacterial and mycoplasmal infections, thereby exacerbating lung lesions and respiratory disease severity [2,41]. In Hungary, a nationwide PRRS eradication programme initiated in 2014 and completed in February 2022 led to official PRRSV-free status [42] and was accompanied by major structural changes in the pig sector, including depopulation–repopulation of many herds [43]. The timing of this programme coincided with the observed decline in EP-like lesions; however, the present study did not include pathogen-level confirmation and therefore cannot establish a causal relationship. The observed improvement may reflect the combined effects of pathogen control, vaccination, improved management and biosecurity, and genetic progress rather than the control of a single infectious agent. The increasing estimated probability of belonging to the structural zero class over time was consistent with the observed increase in zero lesion scores. The reduction in overall mean lung scores was therefore primarily associated with the increasing contribution of structural zeros, while the conditional component did not show a statistically supported consistent temporal decline over the study period. This pattern may be compatible with a decrease in lesion-generating disease pressure at the herd level, rather than merely milder disease expression; however, the latent structural zero class cannot be directly interpreted as evidence of the absence of infection. Similar temporal trends have been described: a statistically significant decrease in the monthly EP index was reported in Spain and Catalonia between 2016 and 2019 [44], supporting the notion that sustained, integrated control measures can lead to measurable improvements in slaughter lung health over time.

### 4.2. Anatomical Distribution of EP-like Lesions

The model indicated significant right–left asymmetry in structural zero probability in most years, with lower probabilities of structural zeros in the right lung. In contrast, differences in the overall predicted lesion scores between lung sides were limited and significant only in 2018. This pattern suggests that anatomical differences in the porcine respiratory tract may contribute to the higher occurrence of lesions in the right lung. This may be related to preferential airflow and aerosol deposition in the right lung [42]; however, these mechanisms were not directly investigated in the present study. The higher involvement of the right lung is consistent with previous slaughterhouse studies reporting a greater prevalence of lesions in right lung lobes [9,13]. Molecular studies have also demonstrated higher detection rates of *Mycoplasma hyopneumoniae* in right lung lobes, particularly the right apical, middle, and caudal lobes [45].

In addition to lung-side differences, the model indicated significant differences among lung lobes in both structural zero probability and the conditional lesion-score component. Cardiac and apical lobes generally showed greater lesion involvement, whereas diaphragmatic and accessory lobes showed lower involvement. A similar cranioventral distribution of lesions has been described in previous studies, with the apical and cardiac lobes being most commonly affected [28,46,47]. However, as no aetiological confirmation was performed in the present study, the observed lesions cannot be attributed to a specific pathogen.

### 4.3. Farm Size in Relation to EP-like Lesions

In the present study, farm size was not significantly associated with the conditional lesion-score component, consistent with previous reports showing no significant association between herd size and lung lesion scores [6,17,48]. The model-estimated structural zero probabilities generally showed an ordering by farm size, with small farms having the lowest estimates and large farms the highest. However, the overall effect of farm size on structural zero probability was not statistically significant, and no significant pairwise differences were detected among farm-size categories. Therefore, this apparent ordering should be interpreted as a pattern in the model estimates rather than as statistical evidence that farm size influenced the probability of belonging to the structural-zero class. Non-infectious factors, such as ventilation, air quality, stocking density, temperature stability, and all-in/all-out (AIAO) management, play an important role in porcine respiratory health [5,16,49]. Farm size may therefore reflect differences in management quality, production intensity, and herd health practices rather than acting as an independent risk factor itself.

### 4.4. Added Value of Raw Means vs. Model-Based Predictions

In zero-inflated slaughterhouse data, raw arithmetic means may obscure epidemiologically important differences. A low mean score may reflect either a high proportion of zero observations or relatively low scores among observations arising from the conditional count process. To address this limitation, some authors have reported means calculated both with and without zero scores [5]. More sophisticated modelling approaches have also been applied in veterinary research, including zero-inflated models for genetic analyses of mastitis case counts in Norwegian Red cows [50] and generalized linear mixed-effects models for the analysis of respiratory disease prevalence in slaughter pigs [39].

The zero-inflated mixed-effects models used in the present study helped separate data components by estimating both the probability of excess zero observations and the expected lesion score from the conditional count process. In the present study, the widening gap between overall predictions incorporating both model components and predictions from the conditional lesion-score component indicated that the temporal decline in overall mean lesion scores was increasingly influenced by observations assigned to the structural zero component rather than solely by changes in the conditional lesion-score distribution. This pattern accompanied the observed increase in the proportion of pigs reaching slaughter without detectable macroscopic EP-like lesions, although the latent structural-zero class should not be interpreted as equivalent to observed lesion absence. This distinction provides a more informative basis for herd-level monitoring than raw means alone.

Sensitivity analyses using alternative representations of the outcome generally yielded qualitatively similar temporal patterns. These analyses should not be interpreted as validation of the primary model, because the ordinal and binomial models rely on their own assumptions and target different aspects of the outcome distribution. Rather, the consistency of the temporal patterns across these analyses suggests that the observed temporal changes were not solely dependent on the specific distributional assumptions of the zero-inflated negative-binomial model.

### 4.5. Limitations

The study used convenience sampling and therefore cannot be regarded as fully representative of the Hungarian pig population [51]. In addition, the examined farms were not identical throughout the study period; consequently, the dataset represents repeated cross-sectional observations rather than a true longitudinal follow-up. Although the sensitivity analysis restricted to repeatedly sampled farms showed a temporal decline similar to that observed in the complete dataset, farm participation remained unbalanced across years. Thus, the observed decline cannot be interpreted as a purely within-farm effect or as definitive evidence of population-wide improvement. Furthermore, although farms from six Hungarian regions and different farm size categories and production systems were included, selection bias cannot be excluded. Therefore, the findings should primarily be interpreted as reflecting the investigated commercial pig population. Future studies using stratified sampling according to geographical region, farm size, and production system would improve population-level representativeness. The absence of microbiological confirmation and detailed information on vaccination, biosecurity, and management practices limits the interpretation of potential causal relationships. Furthermore, macroscopic EP-like lesions are not specific to *Mycoplasma hyopneumoniae* infection and may be associated with multiple respiratory pathogens. Therefore, in the absence of microbiological or molecular confirmation, the observed lesions cannot be used to estimate the prevalence or clinical severity of *M. hyopneumoniae* infection. Slaughterhouse lung scoring primarily reflects the cumulative effects of respiratory disease over the production period and may not adequately capture recent or acute infections. In addition, the interpretation of the zero-inflated component is inherently limited. Structural zeros represent a latent statistical process and cannot be directly equated with the microbiologically confirmed absence of infection or pathogen circulation. The negative-binomial model showed some lack of fit in the upper tail, indicating that the distribution of extreme lesion scores was not fully captured. Simulation-based residual diagnostics also indicated departures from the expected residual distribution, including year- and farm-specific patterns. Although some of these deviations may be accentuated by the large sample size, they indicate remaining model misspecification and should be considered when interpreting model-based estimates. Furthermore, some farm-size-by-year strata contained only a few farms, reducing the precision and robustness of farm-size-specific estimates. The alternative ordinal and binomial sensitivity models also rely on model-specific assumptions, and the categorisation and dichotomisation of Madec scores necessarily result in a loss of information on lesion extent.

## 5. Conclusions

The study provides recent slaughterhouse-based evidence of a marked decline in EP-like lung lesions among sampled Hungarian slaughter pigs between 2018 and 2022. The model-based results indicated that this temporal decline in overall Madec scores was primarily associated with an increasing probability of structural zeros rather than a consistent temporal decline in the conditional lesion-score component. Farm size was not significantly associated with either the conditional lesion-score component or the probability of structural zeros, although the model-estimated structural zero probabilities generally tended to be lower for small farms and higher for large farms in estimable year–farm-size combinations. Lesions showed a characteristic anatomical distribution, with greater lesion involvement in the cardiac and apical lobes and lower structural zero probabilities in the right-sided lobes. Methodologically, the findings illustrate the potential utility of zero-inflated mixed-effects models for analysing slaughterhouse lung-scoring data with a high proportion of zero values and repeated observations. These findings should nevertheless be interpreted as population-level patterns within the sampled commercial pig population rather than as evidence of specific causal mechanisms or pathogen-level changes.

## Figures and Tables

**Figure 1:**
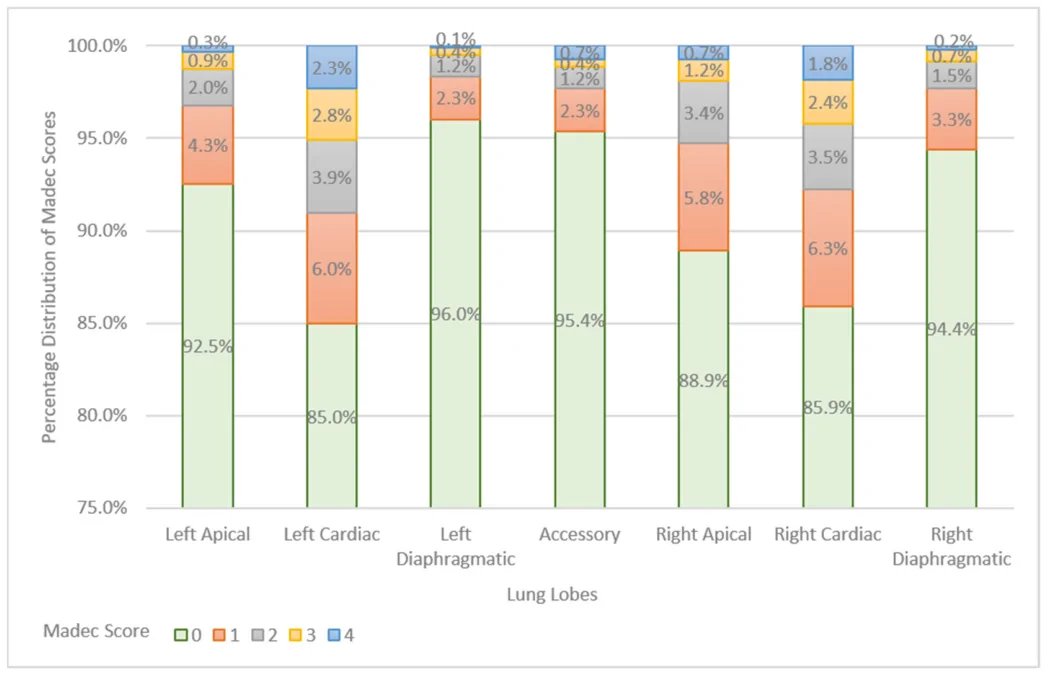
Madec scores among the lung lobes (*n* = 13,499).

**Figure 2:**
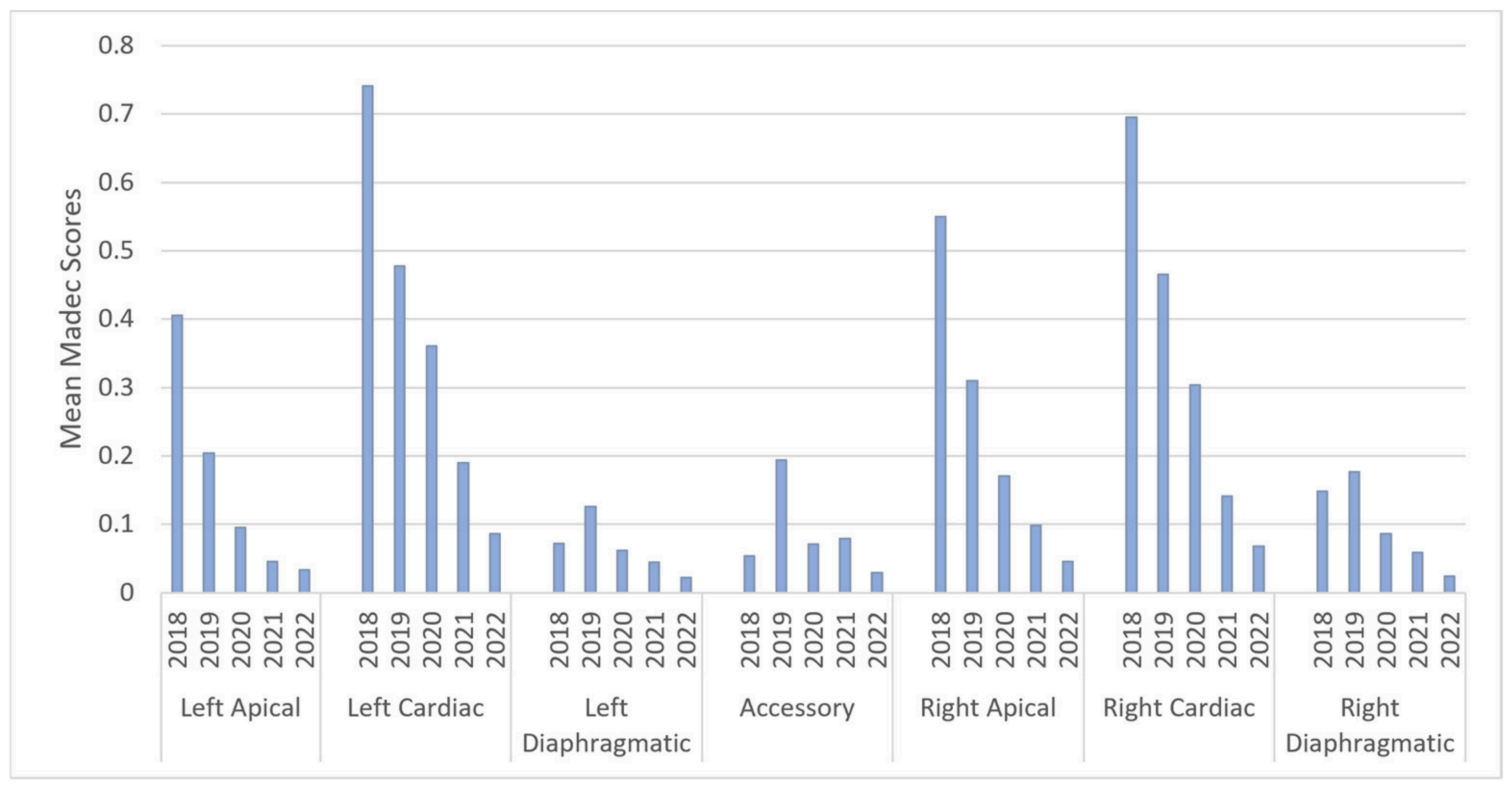
Mean Madec scores by the lung lobes and year (*n* = 13,499).

**Figure 3:**
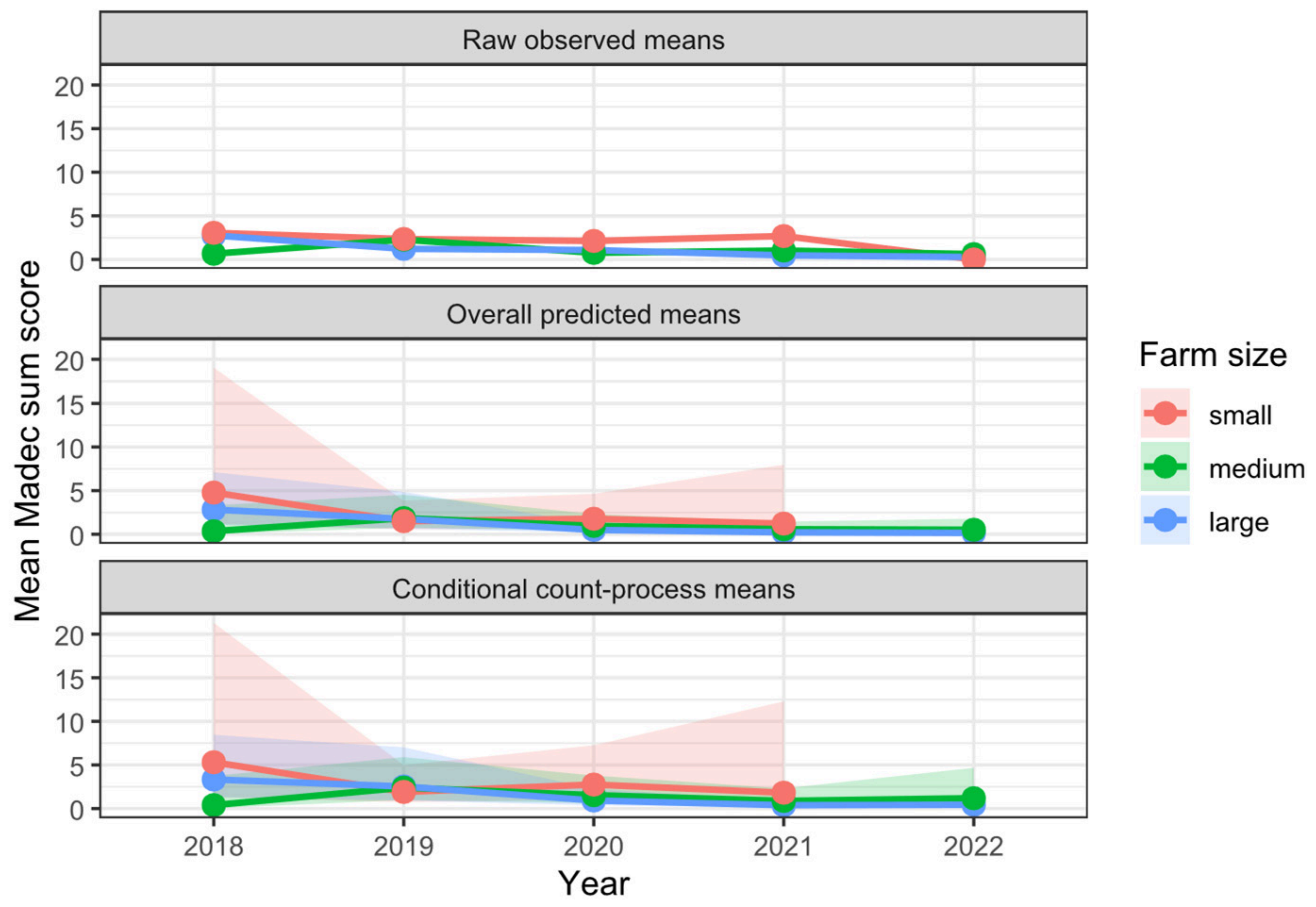
Raw mean Madec sum scores and model-based predicted Madec sum scores by year and farm size. The upper panel shows raw means, the middle panel presents overall predicted means incorporating both model components, and the lower panel shows predicted means from the conditional lesion-score component. Shaded areas represent 95% confidence intervals for model-based predictions. Model-based estimates for small farms in 2022 were not estimable because all observations in this farm-size-year combination had a Madec sum score of zero, preventing reliable estimation of the corresponding model-based values.

**Figure 4:**
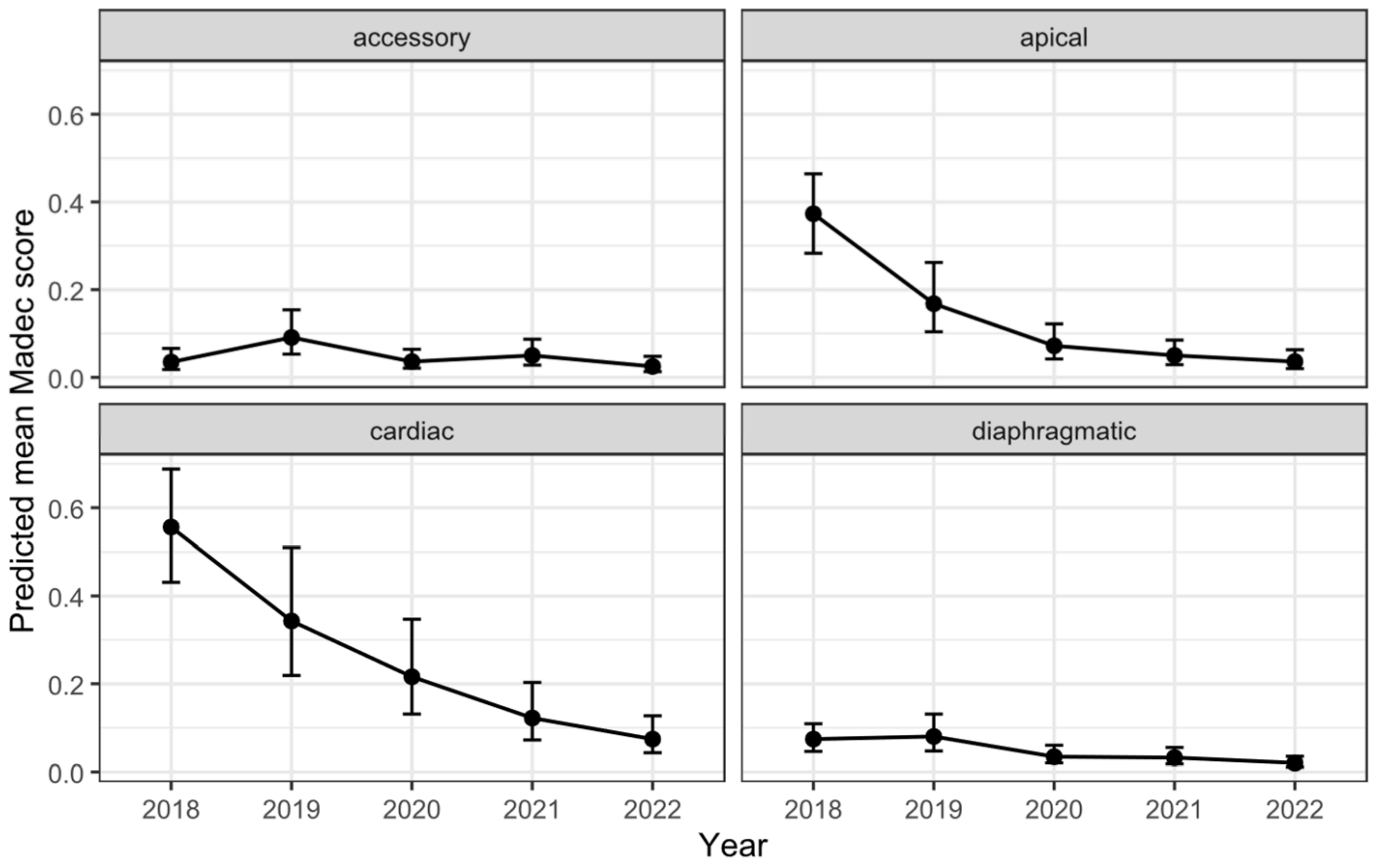
Predicted overall mean Madec scores by year for apical, cardiac, diaphragmatic, and accessory lung lobes. Note: Estimates are derived from the fitted model. Points indicate predicted means and error bars represent 95% confidence intervals.

**Table 1:** Annual distribution of lung examinations.

Year	Farm-Year Observations	Number of Abattoir Examinations	Number of Examined Lungs
2018	8	15	1375
2019	13	34	2817
2020	18	36	2519
2021	16	49	3738
2022	11	40	3086
Total	66	174	13,535

**Table 2:** Summarized Madec scores between 2018–2022. Counts refer to lungs with complete lobe-level data and a calculable Madec sum score.

Year	Lung Count	Madec Score > 0 (Count)	Madec Score > 0 (%)	Mean Madec Score	Madec Score Maximum
2018	1375	789	57.4%	2.7	24
2019	2817	1068	37.9%	2.0	28
2020	2519	701	27.8%	1.1	19
2021	3724	598	16.1%	0.7	22
2022	3064	263	8.6%	0.3	23

**Table 3:** Raw and model-based mean Madec sum scores and probabilities of structural zeros by year and farm size.

Year	2018			2019			2020			2021			2022		
Farm size/ Metric	Small (*N* = 3, *n* = 297)	Med. * (*N* = 1, *n* = 100)	Large (*N* = 4, *n* = 978)	Small (*N* = 6, *n* = 693)	Med. * (*N* = 4, *n* = 1232)	Large (*N* = 3, *n* = 892)	Small (*N* = 7, *n* = 437)	Med. * (*N* = 6, *n* = 863)	Large (*N* = 5, *n* = 1219)	Small (*N* = 2, *n* = 135)	Med. * (*N* = 6, *n* = 743)	Large (*N* = 8, *n* = 2846)	Small (*N* = 2, *n* = 211)	Med. * (*N* = 3, *n* = 443)	Large (*N* = 6, *n* = 2410)
Raw Mean	3.1	0.7	2.8	2.3	2.3	1.2	2.1	0.7	1.1	2.7	1.0	0.5	0.0	0.6	0.3
Prediction (a)	4.8	0.4 **	2.8	1.5	1.8	1.8	1.8	1.0	0.5	1.2 **	0.6	0.2	NE	0.5	0.1
Prediction (b)	5.3	0.4 **	3.3	1.9	2.4	2.5	2.8	1.5	0.9	1.8 **	0.9	0.4	NE	1.2	0.4
Probability of structural zeros	0.100	0.109 **	0.152	0.215	0.231	0.306	0.354	0.375	0.468	0.329 **	0.350	0.441	NE	0.569	0.660

Note: Model-based predictions are shown as (a) overall predicted means incorporating both model components and (b) predicted means from the conditional lesion-score component; *N* = number of distinct farms; *n* = number of examined lungs; * Med. denotes medium-sized farms; ** Small number of farms may limit the precision and reliability of the model-based estimates; NE, not estimable because no lesions were observed in the small-farm 2022 combination, resulting in complete separation and preventing reliable model-based estimation.

## Data Availability

The data presented in this study are available from the corresponding author on reasonable request.

## Supplementary Materials

The following supporting information can be downloaded at: https://www.mdpi.com/article/10.3390/ani16193033/s1, Title: R_script_EP_data_analysis.R; Figure S1: Madec scores distribution among the lung lobes by year; Table S1: Model specification for Madec sum score analysis; Table S2: Comparison of negative-binomial parameterisations for the Madec sum score model using information criteria; Table S3: DHARMa simulation-based residual diagnostics comparing NB2 and NB1 parameterisations for the Madec sum score model; Figure S2: DHARMa residual diagnostics for the final zero-inflated NB1 mixed-effects model for Madec sum score analysis; Figure S3: Normal Q-Q plots of random effects from the final Madec sum score model; Figure S4: DHARMa residual diagnostics for the final Madec sum score model, stratified by year (left) and anonymised farm (right); Figure S5: DHARMa zero-inflation diagnostic for the final Madec sum score model; Table S4: Omnibus Wald χ^2^ tests of fixed effects in the conditional component of the Madec sum score model; Table S5: Omnibus Wald χ^2^ tests of fixed effects in the zero-inflation component of the Madec sum score model; Table S6: Fixed-effect estimates for the conditional component of the Madec sum score analysis; Table S7: Fixed-effect estimates for the zero-inflation component of the Madec sum score analysis; Table S8: Random-effect variance estimates for the Madec sum score analysis; Table S9: Fit statistics for the Madec sum score analysis; Table S10: Overall model-based predicted Madec sum score by year, averaged over farm size categories; Table S11: Overall model-based predicted Madec sum scores, combining the conditional and zero-inflation components; Table S12: Predicted Madec sum scores from the conditional component by year and farm size category; Table S13: Estimated probabilities of structural zeros for the Madec sum score model by year and farm size category; Table S14: Pairwise comparisons of probabilities of structural zeros among farm size categories; Table S15: Predicted Madec sum scores from the conditional component of the sensitivity model restricted to repeatedly sampled farms; Table S16: Overall model-based predicted Madec sum scores, combining the conditional and zero-inflation components, for the sensitivity analysis restricted to repeatedly sampled farms; Table S17: Estimated probabilities of structural zeros for the Madec sum score sensitivity model restricted to repeatedly sampled farms, by year and farm size category; Table S18: Population-level predicted Madec sum score by year for the sensitivity analysis restricted to repeatedly sampled farms, averaged over farm size categories; Table S19: Farm-level cluster-bootstrap sensitivity analysis of farm size contrasts in the final Madec sum score model; Table S20: Model specification for the ordinal sensitivity analysis of the Madec sum score; Table S21: Estimated probabilities of ordinal Madec-score categories by year and farm size category from the cumulative link mixed-effects sensitivity model; Table S22: Model specification for the binary sensitivity analysis of lesion presence; Table S23: Estimated probability of non-zero Madec sum scores by year and farm size category from the binomial mixed-effects sensitivity model; Table S24: Pairwise comparisons of non-zero Madec sum scores among farm size categories within each year from the binomial mixed-effects sensitivity model; Table S25: Omnibus fixed-effect tests under three alternative Madec sum score model specifications; Table S26: Comparison of three alternative Madec sum score model specifications using AIC and BIC; Table S27: Model specifications for the anatomical lobe region analysis; Table S28: Comparison of NB2 and NB1 parameterisations for the anatomical lobe region model using information criteria; Table S29: DHARMa simulation-based residual diagnostics comparing NB2 and NB1 parameterisations for the anatomical lobe region model; Figure S6: DHARMa residual diagnostics for anatomical lobe region analysis; Figure S7: Normal Q-Q plots of random effects from the lobe region model; Figure S8: DHARMa residual diagnostics for the lobe region model, stratified by year (left) and anonymised farm (right); Figure S9: DHARMa zero-inflation diagnostic for the lobe region model; Table S30: Omnibus Wald χ^2^ tests of fixed effects in the conditional component of the anatomical lobe region model; Table S31: Fixed-effect estimates for the conditional component of the anatomical lobe region model; Table S32: Fixed-effect estimates for the zero-inflation component of the anatomical lobe region model; Table S33: Overall model-based predicted lesion grades by anatomical lobe region and year; Table S34: Pairwise comparisons of overall model-based predicted lesion grades among anatomical lobe regions within each year; Table S35: Estimated probabilities of structural zeros for the anatomical lobe region model by lobe region and year; Table S36: Pairwise comparisons of the odds of structural zeros among anatomical lobe regions within each year; Table S37: Model specification for the binary sensitivity analysis of lesion presence by anatomical lobe region; Table S38: Estimated probability of non-zero Madec score by anatomical lobe region within each year from the binomial mixed-effects sensitivity model; Table S39: Pairwise comparisons of lesion presence among anatomical lobe regions within each year from the binomial mixed-effects sensitivity model; Table S40: Model specifications for the left-versus-right lung analysis; Table S41: Comparison of NB2 and NB1 parameterisations for the left-versus-right lung model using information criteria; Table S42: DHARMa simulation-based residual diagnostics comparing NB2 and NB1 parameterisations for the left-versus-right lung model; Figure S10: DHARMa residual diagnostics for the left-versus-right lung model; Figure S11: Normal Q-Q plots of random effects from the left-versus-right lung model; Figure S12: DHARMa residual diagnostics for the left-versus-right lung model, stratified by year (left) and anonymised farm (right); Figure S13: DHARMa zero-inflation diagnostic for the left-versus-right lung model; Table S43: Omnibus Wald χ^2^ tests of fixed effects in the conditional component of the left-versus-right lung model; Table S44: Fixed-effect estimates for the conditional component of the left-versus-right lung model; Table S45: Fixed-effect estimates for the zero-inflation component of the left-versus-right lung model; Table S46: Overall model-based predicted mean Madec scores for the left-versus-right lung model within each year; Table S47: Pairwise comparisons of overall model-based predicted mean Madec scores between the left and right lung sides within each year; Table S48: Estimated probabilities of structural zeros for the left-versus-right lung model by lung side and year; Table S49: Pairwise comparisons of the odds of structural zeros between the left and right lung sides within each year; Table S50: Model specification for the binary sensitivity analysis of lesion presence by lung side; Table S51: Estimated probability of non-zero Madec scores for the left and right lung sides within each year from the binomial mixed-effects sensitivity model; Table S52: Pairwise comparisons of non-zero Madec scores for the left and right lung sides within each year from the binomial mixed-effects sensitivity model.

## Abbreviations

The following abbreviations are used in this manuscript:

EP	Enzootic Pneumonia
PRDC	Porcine Respiratory Disease Complex
PCV2	Porcine Circovirus Type 2
PRRS	Porcine Reproductive and Respiratory Syndrome
QQ Plot	Quantile–Quantile Plot
