# Supplementary material for: Occurrence, Severity and Temporal Trends of Enzootic Pneumonia-like Lung Lesions in Hungarian Slaughter Pigs, 2018–2022: Insights from Zero-Inflated Mixed-Effects Models
Source: Animals (Basel). 2026 Sep 26;16(19):3033. doi: 10.3390/ani16193033 (PMC13663575; doi:10.3390/ani16193033)
Supplement: Supplementary file 1 [file animals-16-03033-s001.zip › animals-4511948-Supplementary-materials.pdf]

Supplementary Materials

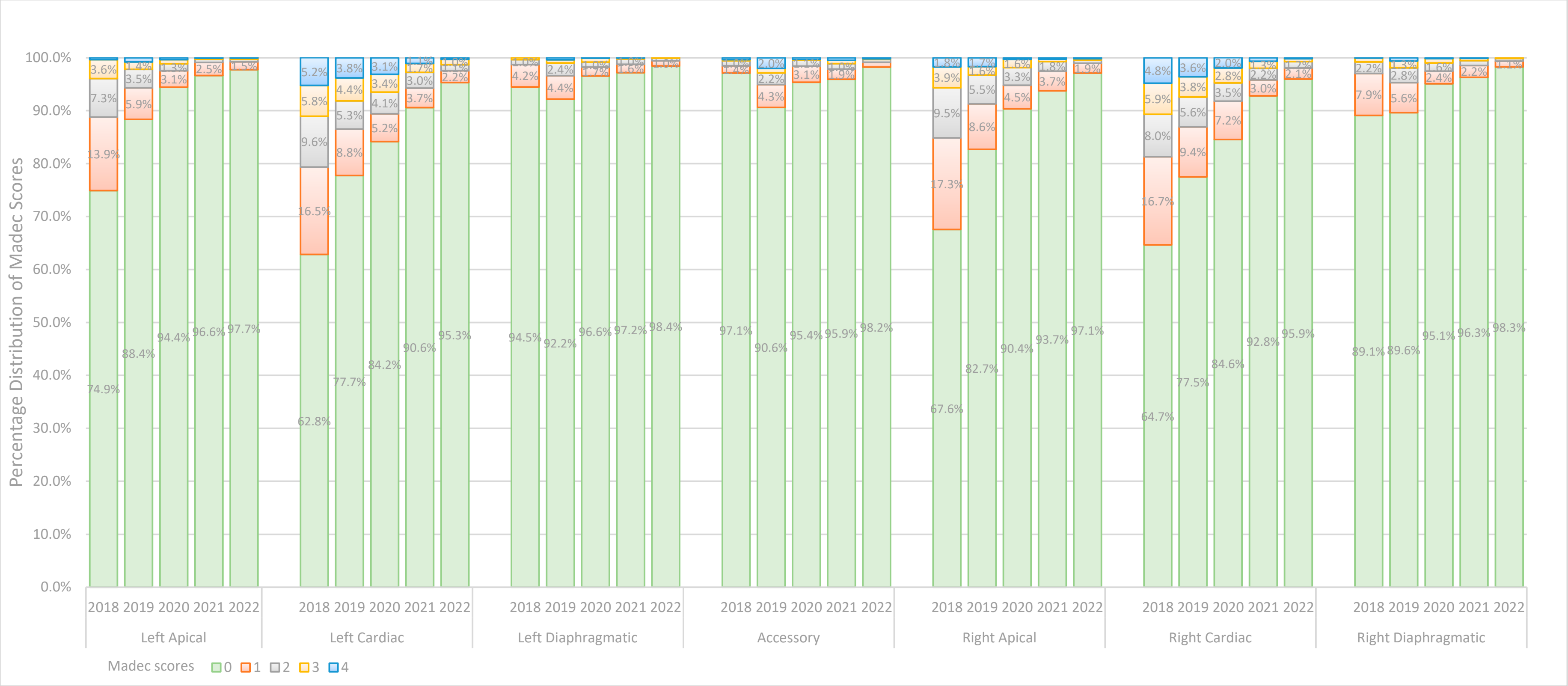

Figure S1: Madec scores distribution among the lung lobes by year. Note: Percentages less than one are not displayed

Table S1: Model specification for Madec sum score analysis

Item	Specification
Response	Total Madec sum score
Distribution and link	Zero-inflated negative binomial (nbinom1), log link
Conditional component	Year × farm size
Zero-inflation component	Year + farm size
Random effects in conditional component	Farm; farm batch nested within farm
Random effects in zero-inflation component	Farm; farm batch nested within farm
Model parametrisation	Conditional component: ~ year × farm size + (1 | farm/farm-batch)<br>Zero-inflation component: ~ year + farm size + (1 | farm/farm-batch)

× indicates interaction between the two variables

Table S2: Comparison of negative-binomial parameterisations for the Madec sum score model using information criteria

Distribution	AIC	BIC
Negative binomial 2 (NB2)	26,881.626	27,084.406
Negative binomial 1 (NB1)	26,849.919	27,052.699

Lower AIC and BIC indicate better relative support. Likelihood-ratio *p*-values are not reported because NB1 and NB2 are non-nested distributional families

Table S3: DHARMa simulation-based residual diagnostics comparing NB2 and NB1 parameterisations for the Madec sum score model

Distribution	Uniformity D	Uniformity *p*-value	Dispersion ratio	Dispersion *p*-value	Zero ratio observed/simulated	Zero-inflation *p*-value
NB2	0.0298	<0.0001	0.493	0.0220	1.041	0.4020
NB1	0.0374	<0.0001	0.075	0.0020	1.035	0.4980

Uniformity is assessed by a Kolmogorov-Smirnov test; dispersion compares observed residual variability with simulations; the zero-inflation test compares observed and simulated numbers of zeros

Model selection conclusion: based on the information criteria, NB1 provided better relative support than NB2 for the Madec sum score model (ΔAIC = 31.7; ΔBIC = 31.7). Simulation-based residual diagnostics indicated some remaining deviations for both parameterisations, with neither model showing evidence of residual zero inflation. Considering the substantially lower AIC and BIC values together with the diagnostic assessment, NB1 was retained for the final Madec sum score analysis.

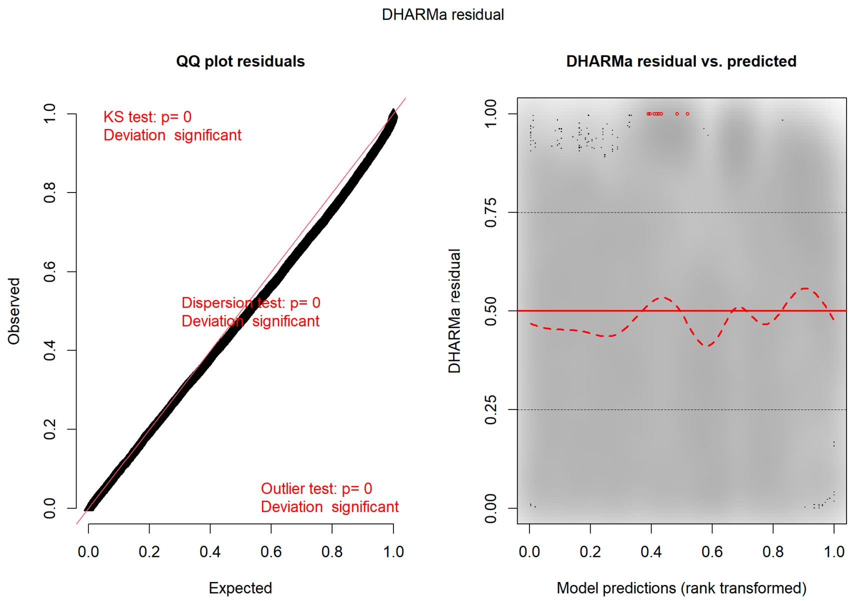

Figure S2: DHARMa residual diagnostics for the final zero-inflated NB1 mixed-effects model for Madec sum score analysis. Note: The Q-Q plot compares simulated residuals with their expected uniform distribution, while the residual-versus-predicted plot shows residuals across rank-transformed fitted values. The Kolmogorov-Smirnov, dispersion and outlier tests indicated significant deviations. Given the large sample size (n = 13,499), the tests have high power to detect small deviations

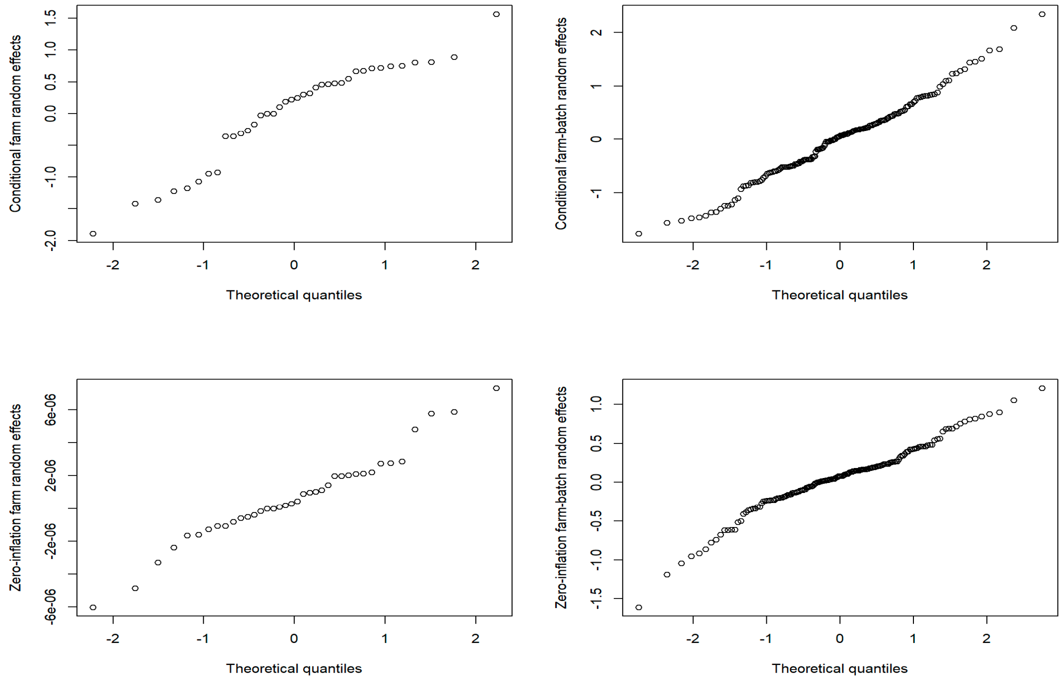

Figure S3: Normal Q-Q plots of random effects from the final Madec sum score model. Note: Panels show farm (upper-left and bottom-left) and farm-batch (upper-right and bottom-right) random effects for the conditional (upper) and zero-inflation (bottom) components

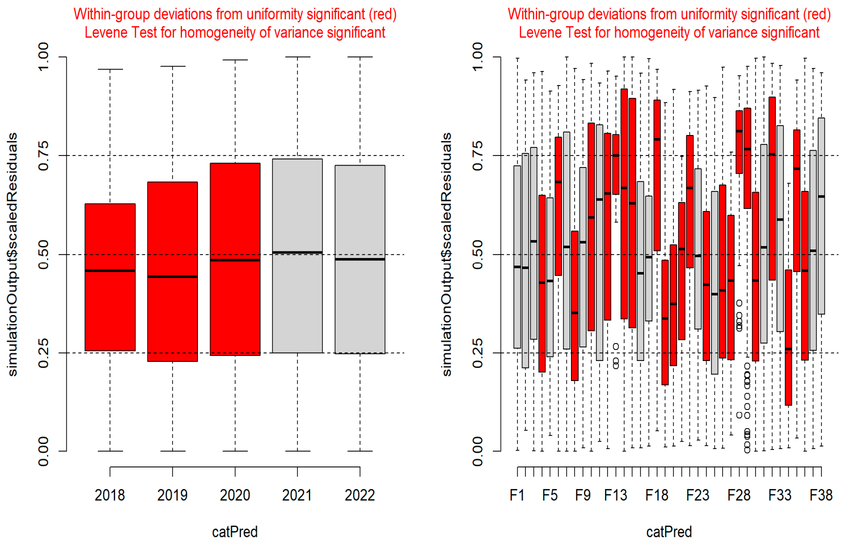

Figure S4: DHARMa residual diagnostics for the final Madec sum score model, stratified by year (left) and anonymised farm (right). Note: Significant within-group deviations from uniformity were detected. Given the large sample size (n = 13,499), the tests are highly sensitive to relatively small deviations

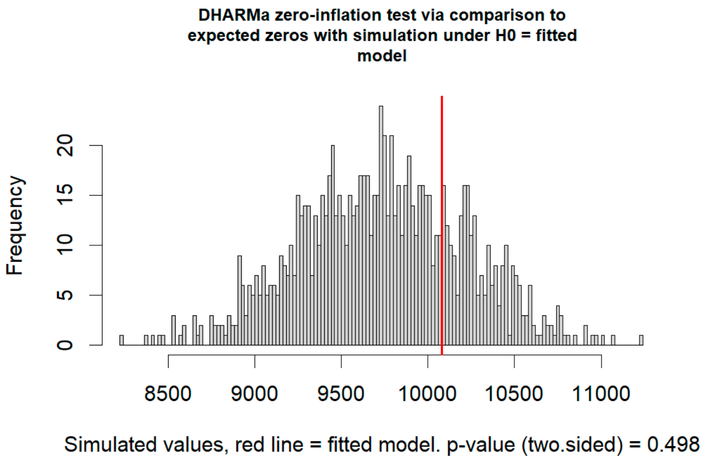

Figure S5: DHARMa zero-inflation diagnostic for the final Madec sum score model. Note: The histogram shows simulated numbers of zeros under the fitted model; the red line shows the observed number of zeros. The DHARMa zero-inflation test was not significant (*p*-value = 0.4980), indicating no evidence that the fitted model inadequately accounted for the observed frequency of zeros

Table S4: Omnibus Wald χ^2 tests of fixed effects in the conditional component of the Madec sum score model. Note: H0: the conditional mean Madec sum score does not differ among the levels of each model effect

Effect	Wald χ^2	df	*p*-value
Year	5.34	4	0.2538
Farm size	0.00	2	0.9985
Year × Farm size	10.53	8	0.2297

Table S5: Omnibus Wald χ^2 tests of fixed effects in the zero-inflation component of the Madec sum score model. Note: H0: the probability of a structural zero does not differ among the levels of each model effect

Effect	Wald χ^2	df	*p*-value
Year	24.19	4	<0.0001
Farm size	3.11	2	0.2112

Table S6: Fixed-effect estimates for the conditional component of the Madec sum score analysis. Note: Estimates are on the log scale

Predictor	Estimate	SE	95% CI	z	*p*-value
(Intercept)	1.203	0.476	0.271 to 2.135	2.53	0.0114
Year 2019	-0.273	0.491	-1.235 to 0.689	-0.56	0.5783
Year 2020	-1.283	0.492	-2.247 to -0.319	-2.61	0.0091
Year 2021	-2.162	0.447	-3.038 to -1.285	-4.83	<0.0001
Year 2022	-2.036	0.530	-3.075 to -0.997	-3.84	0.0001
Farm size medium	-2.138	1.244	-4.577 to 0.301	-1.72	0.0857
Farm size small	0.467	0.852	-1.204 to 2.138	0.55	0.5837
Year 2019 × Farm size medium	2.085	1.277	-0.418 to 4.588	1.63	0.1025
Year 2020 × Farm size medium	2.648	1.243	0.211 to 5.084	2.13	0.0332
Year 2021 × Farm size medium	2.969	1.223	0.571 to 5.366	2.43	0.0152
Year 2022 × Farm size medium	3.142	1.359	0.479 to 5.806	2.31	0.0207
Year 2019 × Farm size small	-0.751	0.880	-2.477 to 0.974	-0.85	0.3934
Year 2020 × Farm size small	0.625	0.928	-1.195 to 2.444	0.67	0.5010
Year 2021 × Farm size small	1.089	1.255	-1.371 to 3.549	0.87	0.3855
Year 2022 × Farm size small	NE	NE	NE	NE	NE

× indicates interaction between the two variables
NE, not estimable, because only two distinct farms were sampled and no lesions were observed

Table S7: Fixed-effect estimates for the zero-inflation component of the Madec sum score analysis. Note: Estimates are on the logit scale

Predictor	Estimate	SE	95% CI	z	*p*-value
(Intercept)	-1.718	0.321	-2.346 to -1.089	-5.35	<0.0001
Year 2019	0.897	0.390	0.133 to 1.661	2.30	0.0214
Year 2020	1.590	0.386	0.834 to 2.347	4.12	<0.0001
Year 2021	1.479	0.430	0.637 to 2.321	3.44	0.0006
Year 2022	2.379	0.538	1.325 to 3.433	4.42	<0.0001
Farm size medium	-0.382	0.292	-0.954 to 0.189	-1.31	0.1898
Farm size small	-0.476	0.304	-1.071 to 0.120	-1.57	0.1176

Table S8: Random-effect variance estimates for the Madec sum score analysis

Component	Group	Parameter	SD	Variance
Conditional	farm-batch:farm	Intercept	0.932	0.869
Conditional	farm	Intercept	0.997	0.993
Zero-inflation	farm-batch:farm	Intercept	0.742	0.551
Zero-inflation	farm	Intercept	0.001	0.000

Table S9: Fit statistics for the Madec sum score analysis

Statistic	Value
AIC	26,849.919
BIC	27,052.699
Log-likelihood	-13,397.960
Residual degrees of freedom	13,472.000
Dispersion parameter	3.084
Observations	13,499.000

Table S10: Overall model-based predicted Madec sum score by year, averaged over farm size categories. Note: The 95% confidence intervals are simulation-based percentile intervals obtained from the joint asymptotic sampling distribution of the conditional and zero-inflation fixed-effect coefficients; random-effects were set to zero; farm-size-specific predictions were averaged within each simulation before the 2.5th and 97.5th percentiles were calculated. The 2022-point estimate is shown because it was obtainable from the fitted model, whereas its corresponding simulation-based confidence interval was not estimable

Year	Predicted mean	95% CI
2018	2.651	1.250 to 7.770
2019	1.702	1.030 to 3.260
2020	1.076	0.627 to 2.160
2021	0.669	0.277 to 2.950
2022	0.220	NE

NE, not estimable

Table S11: Overall model-based predicted Madec sum scores, combining the conditional and zero-inflation components. Note: Point estimates are from emmeans; the 95% confidence intervals are simulation-based and were obtained from the joint asymptotic sampling distribution of the conditional and zero-inflation fixed-effect coefficients; random-effects were set to zero

Year	Farm size	Predicted mean	Simulation-based 95% CI
2018	large	2.823	1.109 to 7.120
2018	medium	0.350	0.036 to 3.313
2018	small	4.779	1.184 to 19.036
2019	large	1.760	0.630 to 4.853
2019	medium	1.848	0.750 to 4.522
2019	small	1.498	0.571 to 3.853
2020	large	0.491	0.202 to 1.170
2020	medium	0.959	0.394 to 2.309
2020	small	1.778	0.667 to 4.629
2021	large	0.214	0.097 to 0.464
2021	medium	0.572	0.220 to 1.451
2021	small	1.220	0.176 to 7.967
2022	large	0.148	0.063 to 0.336
2022	medium	0.511	0.138 to 1.782
2022	small	NE	NE

NE, not estimable, because only two distinct farms were sampled and no lesions were observed

Table S12: Predicted Madec sum scores from the conditional component by year and farm size category

Year	Farm size	Conditional mean	95% CI
2018	large	3.330	1.311 to 8.458
2018	medium	0.392	0.041 to 3.738
2018	small	5.312	1.325 to 21.295
2019	large	2.535	0.915 to 7.018
2019	medium	2.403	0.979 to 5.896
2019	small	1.908	0.731 to 4.981
2020	large	0.923	0.376 to 2.267
2020	medium	1.536	0.623 to 3.783
2020	small	2.751	1.043 to 7.250
2021	large	0.383	0.169 to 0.867
2021	medium	0.879	0.335 to 2.309
2021	small	1.817	0.269 to 12.280
2022	large	0.435	0.163 to 1.158
2022	medium	1.186	0.302 to 4.668
2022	small	NE	NE

NE, not estimable, because only two distinct farms were sampled and no lesions were observed

Table S13: Estimated probabilities of structural zeros for the Madec sum score model by year and farm size category

Year	Farm size	Probability	95% CI
2018	large	0.152	0.087 to 0.252
2018	medium	0.109	0.051 to 0.219
2018	small	0.100	0.050 to 0.191
2019	large	0.306	0.199 to 0.439
2019	medium	0.231	0.148 to 0.342
2019	small	0.215	0.128 to 0.337
2020	large	0.468	0.345 to 0.595
2020	medium	0.375	0.249 to 0.521
2020	small	0.354	0.238 to 0.489
2021	large	0.441	0.310 to 0.580
2021	medium	0.350	0.220 to 0.506
2021	small	0.329	0.190 to 0.506
2022	large	0.660	0.465 to 0.812
2022	medium	0.569	0.359 to 0.758
2022	small	NE	NE

NE, not estimable, because only two distinct farms were sampled and no lesions were observed

Table S14: Pairwise comparisons of probabilities of structural zeros among farm size categories. Note: Odds ratios and Tukey-adjusted *p*-values are shown; because the zero-inflation component included additive effects of year and farm size without a year and farm size interaction, the farm-size contrasts are constant across years

Contrast	Odds ratio	95% CI	Adjusted *p*-value
large / medium	1.466	0.740 to 2.903	0.3889
large / small	1.609	0.789 to 3.281	0.2609
medium / small	1.098	0.496 to 2.432	0.9591

Table S15: Predicted Madec sum scores from the conditional component of the sensitivity model restricted to repeatedly sampled farms

Year	Farm size	Conditional mean	95% CI
2018	large	2.777	0.819 to 9.415
2018	medium	0.337	0.025 to 4.459
2018	small	3.515	0.515 to 23.998
2019	large	2.592	0.794 to 8.456
2019	medium	1.699	0.381 to 7.573
2019	small	1.071	0.261 to 4.392
2020	large	0.781	0.286 to 2.134
2020	medium	1.234	0.313 to 4.871
2020	small	1.572	0.265 to 9.339
2021	large	0.321	0.127 to 0.808
2021	medium	0.782	0.203 to 3.008
2021	small	0.340	0.019 to 6.186
2022	large	0.328	0.121 to 0.887
2022	medium	1.271	0.249 to 6.495
2022	small	NE	NE

NE, not estimable, because only two distinct farms were sampled and no lesions were observed

Table S16: Overall model-based predicted Madec sum scores, combining the conditional and zero-inflation components, for the sensitivity analysis restricted to repeatedly sampled farms. Note: Point estimates are from emmeans; the 95% confidence intervals are simulation-based and were obtained from the joint asymptotic sampling distribution of the conditional and zero-inflation fixed-effect coefficients; random-effects were set to zero

Year	Farm size	Predicted mean	Simulation-based 95% CI
2018	large	2.362	0.691 to 7.892
2018	medium	0.298	0.022 to 3.909
2018	small	3.115	0.450 to 21.047
2019	large	1.505	0.456 to 4.858
2019	medium	1.106	0.248 to 4.846
2019	small	0.701	0.170 to 2.789
2020	large	0.431	0.157 to 1.160
2020	medium	0.770	0.196 to 2.988
2020	small	0.987	0.166 to 5.729
2021	large	0.182	0.072 to 0.449
2021	medium	0.499	0.131 to 1.901
2021	small	0.218	0.012 to 3.822
2022	large	0.127	0.049 to 0.322
2022	medium	0.583	0.113 to 2.896
2022	small	NE	NE

NE, not estimable, because only two distinct farms were sampled and no lesions were observed

Table S17: Estimated probabilities of structural zeros for the Madec sum score sensitivity model restricted to repeatedly sampled farms, by year and farm size category

Year	Farm size	Probability	95% CI
2018	large	0.150	0.080 to 0.264

Year	Farm size	Probability	95% CI
2018	medium	0.116	0.053 to 0.235
2018	small	0.114	0.055 to 0.221
2019	large	0.419	0.314 to 0.533
2019	medium	0.349	0.225 to 0.498
2019	small	0.345	0.223 to 0.493
2020	large	0.448	0.356 to 0.543
2020	medium	0.376	0.260 to 0.509
2020	small	0.372	0.246 to 0.519
2021	large	0.433	0.338 to 0.534
2021	medium	0.362	0.254 to 0.486
2021	small	0.358	0.217 to 0.529
2022	large	0.613	0.486 to 0.727
2022	medium	0.541	0.392 to 0.683
2022	small	NE	NE

NE, not estimable, because only two distinct farms were sampled and no lesions were observed

Table S18: Overall model-based predicted Madec sum score by year for the sensitivity analysis restricted to repeatedly sampled farms, averaged over farm size categories. Note: The 95% confidence intervals are simulation-based percentile intervals obtained from the joint asymptotic sampling distribution of the conditional and zero-inflation fixed-effect coefficients; random-effects were set to zero; farm-size-specific predictions were averaged within each simulation before the 2.5th and 97.5th percentiles were calculated

Year	Predicted mean	Simulation-based 95% CI
2018	1.925	0.769 to 8.464
2019	1.104	0.555 to 2.954
2020	0.729	0.340 to 2.504
2021	0.299	0.133 to 1.609
2022	NE	NE

NE, non-estimable: the 2022 mean and confidence interval were not estimable because no small farms were represented in 2022 in the repeated-farm dataset, resulting in a non-estimable year and farm size interaction term

Table S19: Farm-level cluster-bootstrap sensitivity analysis of farm size contrasts in the final Madec sum score model

Component	Year	Contrast	Measure	Successful replicates (500 attempts)	Bootstrap 95% CI (500 attempts)	Includes 1 (500 attempts)	Successful replicates (665 attempts)	Bootstrap 95% CI (665 attempts)	Includes 1 (665 attempts)
Conditional	2018	large / medium	Conditional mean ratio	403	3.246 to 23.066	No	541	3.045 to 21.926	No
Conditional	2018	large / small	Conditional mean ratio	403	0.237 to 1.803	Yes	541	0.241 to 1.845	Yes
Conditional	2018	medium / small	Conditional mean ratio	403	0.029 to 0.206	No	541	0.029 to 0.207	No
Conditional	2019	large / medium	Conditional mean ratio	403	0.064 to 2.957	Yes	541	0.072 to 3.003	Yes
Conditional	2019	large / small	Conditional mean ratio	403	0.129 to 3.811	Yes	541	0.129 to 4.086	Yes
Conditional	2019	medium / small	Conditional mean ratio	403	0.424 to 6.563	Yes	541	0.392 to 6.552	Yes
Conditional	2020	large / medium	Conditional mean ratio	403	0.249 to 2.584	Yes	541	0.247 to 2.54	Yes
Conditional	2020	large / small	Conditional mean ratio	403	0.123 to 1.032	Yes	541	0.122 to 1.036	Yes
Conditional	2020	medium / small	Conditional mean ratio	403	0.163 to 1.235	Yes	541	0.166 to 1.282	Yes
Conditional	2021	large / medium	Conditional mean ratio	403	0.069 to 4.618	Yes	541	0.063 to 4.116	Yes
Conditional	2021	large / small	Conditional mean ratio	403	0.024 to 1.719	Yes	541	0.024 to 1.718	Yes
Conditional	2021	medium / small	Conditional mean ratio	403	0.021 to 2.882	Yes	541	0.021 to 3.828	Yes
Conditional	2022	large / medium	Conditional mean ratio	403	0.098 to 5.764	Yes	541	0.101 to 6.485	Yes
Conditional	2022	large / small	Conditional mean ratio	403	NE	NE	541	NE	NE
Conditional	2022	medium / small	Conditional mean ratio	403	NE	NE	541	NE	NE
Zero-inflation	-	large / medium	Odds ratio	403	0.769 to 4.011	Yes	541	0.773 to 4.057	Yes
Zero-inflation	-	large / small	Odds ratio	403	0.703 to 3.344	Yes	541	0.695 to 3.55	Yes
Zero-inflation	-	medium / small	Odds ratio	403	0.298 to 2.418	Yes	541	0.284 to 2.369	Yes

Farms were resampled with replacement, and the final model was refitted to each bootstrap sample. A total of 500 bootstrap resampling attempts were performed, of which 403 resulted in successful model fits and were retained for the analysis. As a sensitivity analysis, the number of bootstrap resampling attempts was increased to 665, of which 541 resulted in successful model fits. Percentile 95% bootstrap confidence intervals are shown for conditional mean ratios in the conditional component and odds ratios in the zero-inflation component. Intervals including 1 do not provide evidence of a farm size difference. NE: not estimable as small farms in 2022 were retained. The zero-inflation farm size contrasts are reported once because the final zero-inflation model contains no year and farm size interaction

Conclusion for farm-level cluster bootstrap sensitivity analysis of farm size contrasts: the farm-level cluster bootstrap broadly supported the primary model results, with no consistent evidence of farm size differences. Most 95% confidence intervals included 1; exceptions were limited to two conditional contrasts in 2018 (large/medium and medium/small), while all zero-inflation contrasts included 1.

In the original analysis, 403 of 500 attempted resamples produced usable model fits; 96 unsuccessful fits had a non-positive-definite Hessian and one failed because of an NA/NaN (Not Available/ Not a Number) gradient evaluation. As a sensitivity analysis, we increased the number of attempted

farm-level bootstrap resamples from 500 to 665, which increased the number of usable model fits from 403 to 541. In the sensitivity analysis, 541 of 665 attempted resamples produced usable model fits; 123 unsuccessful fits had a non-positive-definite Hessian and one failed because of an NA/NaN (Not Available/ Not a Number) gradient evaluation. The resulting percentile 95% confidence intervals were very similar to those obtained from the original 403 usable fits, and the interpretation with respect to inclusion of the null value (1) was unchanged for all estimable contrasts. Thus, the exclusion of unsuccessful replicates did not materially affect the bootstrap results, as the confidence intervals remained very similar when the number of usable fits increased from 403 to 541.

Table S20: Model specification for the ordinal sensitivity analysis of the Madec sum score. Note: The Madec sum score was analysed as an ordered three-category outcome (0, 1-4, and 5-28); small farms in 2022 were excluded because this combination did not support reliable estimation

**Item**	**Specification**
Response	Madec sum score categorized as 0, 1-4, and 5-28 (ordered outcome)
Model	Cumulative link mixed-effects model
Link	Logit
Threshold structure	Flexible
Fixed effects	Year × farm size
Random effects	Farm; farm batch nested within farm
Estimation	Laplace approximation (nAGQ = 1)
Excluded combination	Small farms in 2022

× indicates interaction between the two variables

Table S21: Estimated probabilities of ordinal Madec-score categories by year and farm size category from the cumulative link mixed-effects sensitivity model. Note: 95% confidence intervals were calculated on the logit-probability scale using the delta method and back-transformed to the probability scale

Year	Farm size	Score category	Probability	SE	95% CI
2018	large	0	0.382	0.125	0.180 to 0.635
2018	medium	0	0.868	0.146	0.350 to 0.988
2018	small	0	0.217	0.135	0.055 to 0.569
2019	large	0	0.560	0.140	0.295 to 0.795
2019	medium	0	0.510	0.127	0.278 to 0.738
2019	small	0	0.536	0.135	0.285 to 0.770
2020	large	0	0.818	0.073	0.633 to 0.921
2020	medium	0	0.680	0.108	0.446 to 0.848
2020	small	0	0.606	0.130	0.346 to 0.818
2021	large	0	0.916	0.034	0.822 to 0.962
2021	medium	0	0.792	0.086	0.578 to 0.913
2021	small	0	0.621	0.247	0.173 to 0.928
2022	large	0	0.946	0.024	0.876 to 0.977
2022	medium	0	0.794	0.111	0.504 to 0.936
2022	small	0	NE	NE	NE
2018	large	1-4	0.385	0.031	0.326 to 0.447
2018	medium	1-4	0.104	0.112	0.011 to 0.549
2018	small	1-4	0.379	0.057	0.276 to 0.495
2019	large	1-4	0.311	0.076	0.184 to 0.476
2019	medium	1-4	0.337	0.061	0.229 to 0.466
2019	small	1-4	0.324	0.070	0.204 to 0.473
2020	large	1-4	0.142	0.054	0.065 to 0.283
2020	medium	1-4	0.239	0.071	0.128 to 0.402
2020	small	1-4	0.285	0.077	0.159 to 0.456
2021	large	1-4	0.067	0.026	0.031 to 0.141
2021	medium	1-4	0.161	0.062	0.072 to 0.322

Year	Farm size	Score category	Probability	SE	95% CI
2021	small	1-4	0.276	0.150	0.081 to 0.625
2022	large	1-4	0.044	0.019	0.019 to 0.100
2022	medium	1-4	0.159	0.081	0.055 to 0.383
2022	small	1-4	NE	NE	NE
2018	large	5-28	0.233	0.094	0.097 to 0.461
2018	medium	5-28	0.028	0.035	0.002 to 0.259
2018	small	5-28	0.404	0.191	0.125 to 0.763
2019	large	5-28	0.128	0.064	0.046 to 0.310
2019	medium	5-28	0.153	0.066	0.063 to 0.329
2019	small	5-28	0.140	0.065	0.053 to 0.321
2020	large	5-28	0.040	0.019	0.016 to 0.099
2020	medium	5-28	0.081	0.037	0.032 to 0.189
2020	small	5-28	0.109	0.053	0.040 to 0.262
2021	large	5-28	0.017	0.007	0.007 to 0.039
2021	medium	5-28	0.047	0.023	0.017 to 0.121
2021	small	5-28	0.103	0.097	0.014 to 0.473
2022	large	5-28	0.011	0.005	0.004 to 0.026
2022	medium	5-28	0.046	0.030	0.013 to 0.156
2022	small	5-28	NE	NE	NE

NE, not estimable

Conclusion of the ordinal sensitivity analysis: the ordinal model broadly supported the temporal pattern observed with the primary analysis, showing an increasing probability of a zero Madec score and decreasing probabilities of higher score categories over time. Although the magnitude of these patterns varied among farm size categories, the sensitivity analysis did not indicate a materially different overall interpretation from the primary analysis.

Table S22: Model specification for the binary sensitivity analysis of lesion presence. Note: The binary outcome indicates whether a macroscopic EP-like lesion was present; small farms in 2022 were excluded to match the ordinal sensitivity dataset

**Item**	**Specification**
Response	Lesion presence (binary: present versus absent)
Model	Generalized linear mixed-effects model
Distribution	Binomial
Link	Logit
Fixed effects	Year × farm size
Random effects	Farm; farm batch nested within farm
Excluded combination	Small farms in 2022

× indicates interaction between the two variables

Table S23: Estimated probability of non-zero Madec sum scores by year and farm size category from the binomial mixed-effects sensitivity model. Note: Probabilities and 95% confidence intervals are back-transformed from the logit scale

Year	Farm size	Probability	SE	95% CI
2018	large	0.625	0.125	0.370 to 0.826
2018	medium	0.134	0.149	0.013 to 0.656
2018	small	0.823	0.117	0.490 to 0.958
2019	large	0.430	0.140	0.197 to 0.698
2019	medium	0.476	0.128	0.249 to 0.713
2019	small	0.498	0.138	0.252 to 0.745
2020	large	0.175	0.071	0.074 to 0.359
2020	medium	0.324	0.109	0.152 to 0.560
2020	small	0.384	0.130	0.175 to 0.647
2021	large	0.083	0.034	0.037 to 0.177
2021	medium	0.193	0.082	0.079 to 0.401
2021	small	0.348	0.240	0.063 to 0.810
2022	large	0.053	0.024	0.022 to 0.123
2022	medium	0.202	0.110	0.062 to 0.492
2022	small	NE	NE	NE

NE, not estimable

Table S24: Pairwise comparisons of non-zero Madec sum scores among farm size categories within each year from the binomial mixed-effects sensitivity model. Note: Tukey-adjusted *p*-values are shown

Year	Contrast	Odds ratio	95% CI	Adjusted *p*-value
2018	large / medium	10.754	0.418 to 276.634	0.1998
2018	large / small	0.359	0.037 to 3.456	0.5388
2018	medium / small	0.033	0.001 to 1.168	0.0644
2019	large / medium	0.831	0.137 to 5.058	0.9687
2019	large / small	0.759	0.118 to 4.886	0.9358
2019	medium / small	0.913	0.156 to 5.336	0.9920
2020	large / medium	0.443	0.085 to 2.300	0.4781
2020	large / small	0.340	0.060 to 1.923	0.3106

Year	Contrast	Odds ratio	95% CI	Adjusted p-value
2020	medium / small	0.767	0.134 to 4.380	0.9320
2021	large / medium	0.379	0.076 to 1.890	0.3332
2021	large / small	0.169	0.012 to 2.484	0.2679
2021	medium / small	0.447	0.028 to 7.122	0.7743
2022	large / medium	0.223	0.044 to 1.118	0.0681
2022	large / small	NE	NE	NE
2022	medium / small	NE	NE	NE

NE, not estimable

Conclusions from the binomial sensitivity analysis: no statistically significant pairwise differences in lesion presence were detected among farm size categories within any year after adjustment for multiple comparisons. This broadly supports the primary analysis, which likewise provided no consistent evidence of differences among farm size categories.

Table S25: Omnibus fixed-effect tests under three alternative Madec sum score model specifications. Note: The Model column gives the complete conditional and zero-inflation specification for each model

Model	Component	Effect	Wald χ^2	df	p-value
Original - NB2: conditional = year + farm size + (1 | farm/farm-batch); ZI = year × farm size	Conditional	Year	29.952	4	<0.0001
Original - NB2: conditional = year + farm size + (1 | farm/farm-batch); ZI = year × farm size	Conditional	Farm size	1.830	2	0.4006
Revised - NB2: conditional = year × farm size + (1 | farm-batch); ZI = year + farm size + (1 | farm-batch)	Conditional	Year	0.556	4	0.9679
Revised - NB2: conditional = year × farm size + (1 | farm-batch); ZI = year + farm size + (1 | farm-batch)	Conditional	Farm size	0.218	2	0.8964
Revised - NB2: conditional = year × farm size + (1 | farm-batch); ZI = year + farm size + (1 | farm-batch)	Conditional	Year × Farm size	8.528	8	0.3840
Final - NB1: conditional = year × farm size + (1 | farm/farm-batch); ZI = year + farm size + (1 | farm/farm-batch)	Conditional	Year	5.344	4	0.2538
Final - NB1: conditional = year × farm size + (1 | farm/farm-batch); ZI = year + farm size + (1 | farm/farm-batch)	Conditional	Farm size	0.004	2	0.9985
Final - NB1: conditional = year × farm size + (1 | farm/farm-batch); ZI = year + farm size + (1 | farm/farm-batch)	Conditional	Year × Farm size	10.528	8	0.2297
Original - NB2: conditional = year + farm size + (1 | farm/farm-batch); ZI = year × farm size	Zero-inflation	Year	26.928	4	<0.0001
Original - NB2: conditional = year + farm size + (1 | farm/farm-batch); ZI = year × farm size	Zero-inflation	Farm size	0.008	2	0.9962
Original - NB2: conditional = year + farm size + (1 | farm/farm-batch); ZI = year × farm size	Zero-inflation	Year × Farm size	58.512	8	<0.0001
Revised - NB2: conditional = year × farm size + (1 | farm-batch); ZI = year + farm size + (1 | farm-batch)	Zero-inflation	Year	63.652	4	<0.0001
Revised - NB2: conditional = year × farm size + (1 | farm-batch); ZI = year + farm size + (1 | farm-batch)	Zero-inflation	Farm size	6.004	2	0.0497
Final - NB1: conditional = year × farm size + (1 | farm/farm-batch); ZI = year + farm size + (1 | farm/farm-batch)	Zero-inflation	Year	24.188	4	<0.0001
Final - NB1: conditional = year × farm size + (1 | farm/farm-batch); ZI = year + farm size + (1 | farm/farm-batch)	Zero-inflation	Farm size	3.110	2	0.2112

Conclusions from the sensitivity analysis of alternative Madec sum score model specifications: the comparison showed that some statistical conclusions were sensitive to model specification. The year effect in the zero-inflation component was consistently significant across all three specifications, whereas the conditional year effect was significant only in the original model. Farm size effects were also specification-dependent.

Table S26: Comparison of three alternative Madec sum score model specifications using AIC and BIC. Note: All three models were fitted to the same observations; lower AIC and BIC indicate better relative support; Delta AIC and Delta BIC are calculated relative to the lowest value among the three specifications

Model	Family	n	AIC	Delta AIC	BIC	Delta BIC
Original - NB2: conditional = year + farm size + (1 | farm/farm-batch); ZI = year × farm size	NB2	13,499	27,403.534	553.615	27,591.293	538.594
Revised - NB2: conditional = year × farm size + (1 | farm-batch); ZI = year + farm size + (1 | farm-batch)	NB2	13,499	26,959.428	109.509	27,147.187	94.488
Final - NB1: conditional = year × farm size + (1 | farm/farm-batch); ZI = year + farm size + (1 | farm/farm-batch)	NB1	13,499	26,849.919	0.000	27,052.699	0.000

Table S27: Model specifications for the anatomical lobe region analysis

Item	Specification
Response	Lobe-specific lesion grade
Distribution and link	Zero-inflated negative binomial (nbinom2), log link
Conditional component	Lobe region × year + farm size
Zero-inflation component	Year × lobe region
Random effects in conditional component	Farm; animal ID nested within farm
Random effects in zero-inflation component	Farm; animal ID nested within farm
Model parametrisation	Conditional component: ~ lobe region × year + farm size + (1 | farm/animal ID)<br>Zero-inflation component: ~ lobe region × year + (1 | farm/animal ID)

× indicates interaction between the two variables

Table S28: Comparison of NB2 and NB1 parameterisations for the anatomical lobe region model using information criteria

Distribution	AIC	BIC
Negative binomial 2 (NB2)	62,904.736	63,349.181
Negative binomial 1 (NB1)	62,904.736	63,349.181

Lower AIC and BIC indicate better relative support. NB1 and NB2 are non-nested distributional families; therefore, likelihood-ratio *p*-values are not reported

Table S29: DHARMa simulation-based residual diagnostics comparing NB2 and NB1 parameterisations for the anatomical lobe region model

Distribution	Uniformity D	Uniformity *p*-value	Dispersion ratio	Dispersion *p*-value	Zero ratio observed/simulated	Zero-inflation *p*-value
NB2	0.0246	<0.0001	0.536	0.0520	1.030	0.3980
NB1	0.0248	<0.0001	0.526	0.0460	1.033	0.4100

Uniformity is assessed by a Kolmogorov-Smirnov test; dispersion compares observed residual variability with simulations; the zero-inflation test compares observed and simulated numbers of zeros

Model selection conclusion: AIC and BIC were identical for the NB1 and NB2 parameterisations. Based on the overall residual diagnostic assessment, NB2 was retained for the final anatomical lobe-region model.

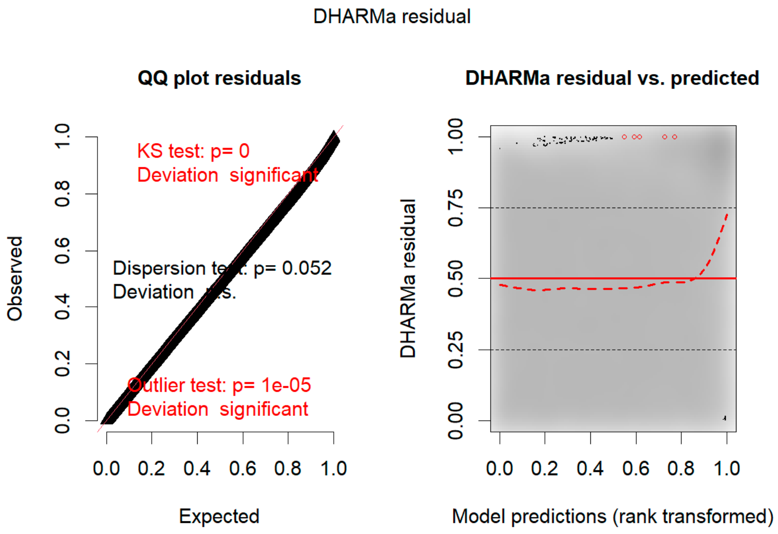

Figure S6: DHARMa residual diagnostics for anatomical lobe region analysis. Note: The Q-Q plot compares simulated residuals with their expected uniform distribution, while the residual-versus-predicted plot shows residuals across rank-transformed fitted values. The Kolmogorov-Smirnov and outlier tests indicated significant deviations. Given the large number of observations used in the model (n = 94,493), the tests have high power to detect small deviations

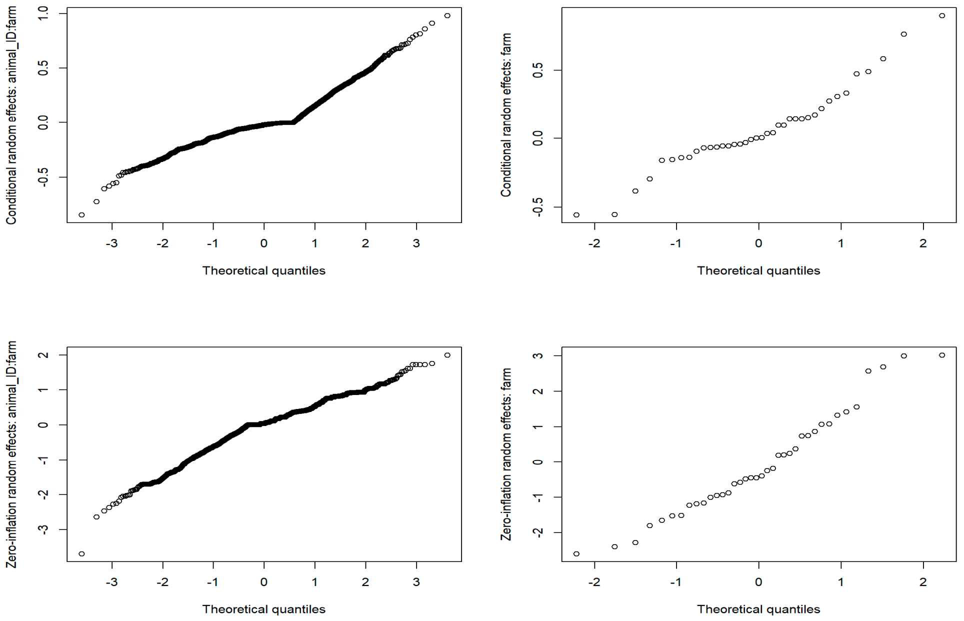

Figure S7: Normal Q-Q plots of random effects from the lobe region model. Note: Panels show animal-ID (upper-left and bottom-left) and farm (upper-right and bottom-right) random effects for the conditional (upper) and zero-inflation (bottom) components

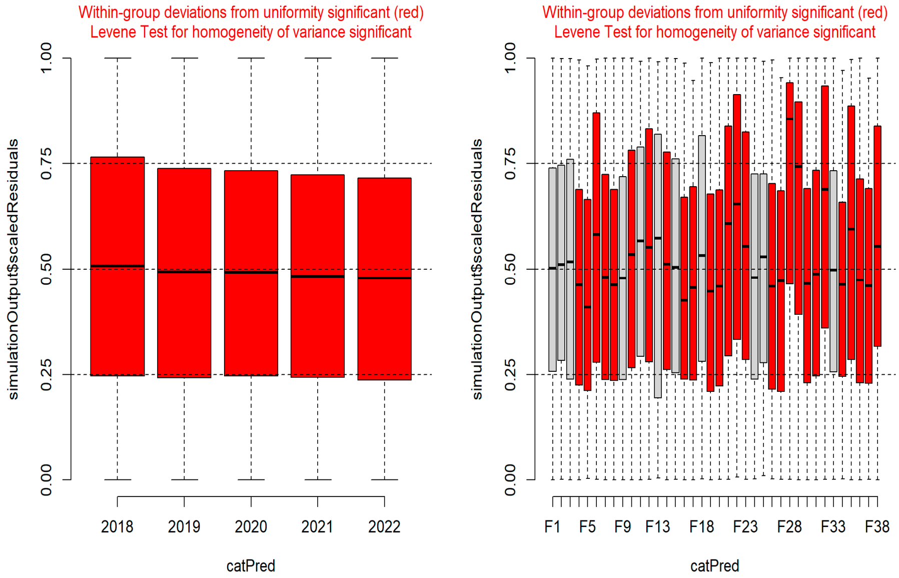

Figure S8: DHARMa residual diagnostics for the lobe region model, stratified by year (left) and anonymised farm (right). Note: Significant within-group deviations from uniformity were detected. Given the large sample size (n = 94,493), the tests are highly sensitive to relatively small deviations

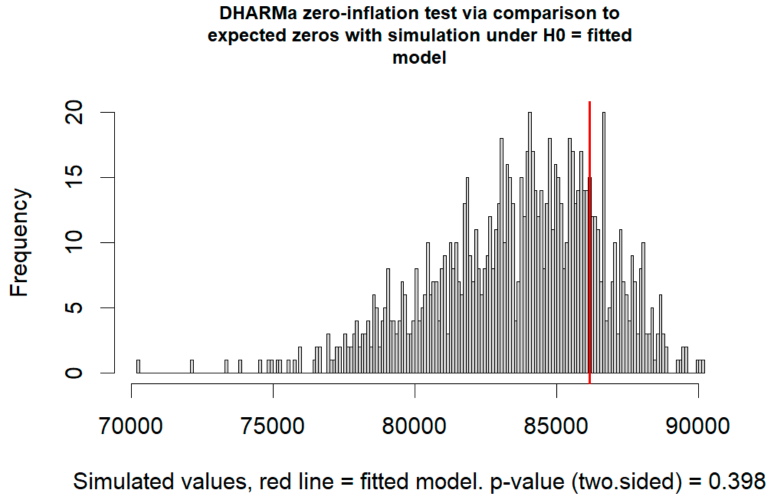

Figure S9: DHARMa zero-inflation diagnostic for the lobe region model. Note: The histogram shows simulated numbers of zeros under the fitted model; the red line shows the observed number of zeros. The DHARMa zero-inflation test was not significant (p-value = 0.3980), indicating no evidence that the fitted model inadequately accounted for the observed frequency of zeros

Table S30: Omnibus Wald χ^2 tests of fixed effects in the conditional component of the anatomical lobe region model. Note: H0: the conditional mean lobe score does not differ among the levels of each model effect

Effect	Wald χ^2	df	p-value
Lobe region	282.24	3	<0.0001
Year	59.72	4	<0.0001

Effect	Wald χ^2	df	*p*-value
Farm size	2.81	2	0.2459
Lobe region × year	84.16	12	<0.0001

Table S31: Fixed-effect estimates for the conditional component of the anatomical lobe region model. Note: Estimates are on the log scale

Predictor	Estimate	SE	95% CI	z	*p*-value
(Intercept)	0.127	0.205	-0.275 to 0.528	0.62	0.5368
Lobe region apical	-0.696	0.168	-1.025 to -0.366	-4.14	<0.0001
Lobe region cardiac	-0.333	0.167	-0.660 to -0.007	-2.00	0.0456
Lobe region diaphragmatic	-1.528	0.197	-1.914 to -1.142	-7.76	<0.0001
Year 2019	0.001	0.175	-0.341 to 0.343	0.00	0.9970
Year 2020	-0.406	0.216	-0.830 to 0.017	-1.88	0.0599
Year 2021	0.294	0.197	-0.092 to 0.679	1.49	0.1356
Year 2022	0.175	0.250	-0.315 to 0.665	0.70	0.4831
Farm size medium	-0.103	0.156	-0.408 to 0.203	-0.66	0.5100
Farm size small	0.143	0.153	-0.157 to 0.443	0.94	0.3489
Lobe region apical × year2019	0.494	0.181	0.139 to 0.850	2.73	0.0064
Lobe region cardiac × year2019	0.508	0.178	0.160 to 0.856	2.86	0.0042
Lobe region diaphragmatic × year2019	1.168	0.211	0.754 to 1.582	5.53	<0.0001
Lobe region apical × year2020	1.029	0.221	0.595 to 1.463	4.65	<0.0001
Lobe region cardiac × year2020	1.075	0.215	0.653 to 1.498	4.99	<0.0001
Lobe region diaphragmatic × year2020	1.844	0.249	1.357 to 2.332	7.42	<0.0001
Lobe region apical × year2021	0.036	0.201	-0.357 to 0.429	0.18	0.8580
Lobe region cardiac × year2021	0.330	0.190	-0.043 to 0.702	1.74	0.0826
Lobe region diaphragmatic × year2021	1.037	0.227	0.592 to 1.482	4.57	<0.0001
Lobe region apical × year2022	0.342	0.252	-0.151 to 0.836	1.36	0.1736
Lobe region cardiac × year2022	0.329	0.237	-0.135 to 0.793	1.39	0.1647
Lobe region diaphragmatic × year2022	0.862	0.289	0.296 to 1.428	2.99	0.0028

× indicates interaction between the two variables

Table S32: Fixed-effect estimates for the zero-inflation component of the anatomical lobe region model. Note: Estimates are on the logit scale

Predictor	Estimate	SE	95% CI	z	*p*-value
(Intercept)	3.480	0.341	2.812 to 4.149	10.20	<0.0001
Year 2019	-1.022	0.221	-1.455 to -0.589	-4.62	<0.0001
Year 2020	-0.471	0.260	-0.981 to 0.040	-1.81	0.0709
Year 2021	-0.077	0.234	-0.536 to 0.382	-0.33	0.7430
Year 2022	0.506	0.278	-0.039 to 1.052	1.82	0.0689
Lobe region apical	-4.082	0.225	-4.524 to -3.641	-18.12	<0.0001
Lobe region cardiac	-4.195	0.216	-4.619 to -3.771	-19.39	<0.0001
Lobe region diaphragmatic	-2.602	0.264	-3.119 to -2.084	-9.85	<0.0001
Year 2019 × Lobe region apical	3.154	0.252	2.661 to 3.648	12.52	<0.0001
Year 2020 × Lobe region apical	3.701	0.287	3.138 to 4.263	12.90	<0.0001
Year 2021 × Lobe region apical	3.390	0.263	2.876 to 3.905	12.90	<0.0001

Predictor	Estimate	SE	95% CI	z	*p*-value
Year 2022 × Lobe region apical	3.354	0.306	2.754 to 3.953	10.97	<0.0001
Year 2019 × Lobe region cardiac	2.842	0.240	2.372 to 3.312	11.85	<0.0001
Year 2020 × Lobe region cardiac	3.053	0.275	2.515 to 3.592	11.11	<0.0001
Year 2021 × Lobe region cardiac	3.249	0.245	2.769 to 3.729	13.27	<0.0001
Year 2022 × Lobe region cardiac	3.073	0.287	2.511 to 3.636	10.71	<0.0001
Year 2019 × Lobe region diaphragmatic	2.356	0.291	1.787 to 2.926	8.11	<0.0001
Year 2020 × Lobe region diaphragmatic	2.982	0.323	2.350 to 3.615	9.25	<0.0001
Year 2021 × Lobe region diaphragmatic	2.550	0.298	1.966 to 3.133	8.57	<0.0001
Year 2022 × Lobe region diaphragmatic	2.178	0.351	1.490 to 2.867	6.20	<0.0001

× indicates interaction between the two variables

Table S33: Overall model-based predicted lesion grades by anatomical lobe region and year. Note: Predicted means combine the conditional and zero-inflation components and are averaged equally over farm size categories; point estimates are from emmeans; 95% confidence intervals are simulation-based and use the joint covariance of conditional and zero-inflation fixed effects; random-effects were set to zero

Year	Lobe type	Predicted mean	Simulation-based 95% CI
2018	accessory	0.035	0.018 to 0.067
2018	apical	0.373	0.285 to 0.466
2018	cardiac	0.556	0.432 to 0.688
2018	diaphragmatic	0.074	0.046 to 0.110
2019	accessory	0.091	0.053 to 0.155
2019	apical	0.168	0.104 to 0.264
2019	cardiac	0.343	0.220 to 0.515
2019	diaphragmatic	0.080	0.047 to 0.132
2020	accessory	0.036	0.020 to 0.064
2020	apical	0.072	0.043 to 0.122
2020	cardiac	0.216	0.132 to 0.348
2020	diaphragmatic	0.034	0.020 to 0.060
2021	accessory	0.050	0.028 to 0.088
2021	apical	0.050	0.029 to 0.084
2021	cardiac	0.122	0.072 to 0.203
2021	diaphragmatic	0.032	0.018 to 0.056
2022	accessory	0.025	0.013 to 0.048
2022	apical	0.036	0.020 to 0.063
2022	cardiac	0.074	0.043 to 0.127
2022	diaphragmatic	0.020	0.011 to 0.035

Table S34: Pairwise comparisons of overall model-based predicted lesion grades among anatomical lobe regions within each year. Note: Comparisons are on the overall response scale and are averaged over farm size categories; random-effects were set to zero; pairwise differences, 95% confidence intervals, and Tukey-adjusted *p*-values were obtained using emmeans

Year	Contrast	Difference	95% CI	Adjusted *p*-value
2018	apical vs accessory	0.338	0.067 to 0.609	0.0073
2018	apical vs cardiac	-0.184	-0.332 to -0.035	0.0080

Year	Contrast	Difference	95% CI	Adjusted *p*-value
2018	apical vs diaphragmatic	0.299	0.077 to 0.521	0.0030
2018	cardiac vs accessory	0.522	0.112 to 0.932	0.0059
2018	cardiac vs diaphragmatic	0.483	0.122 to 0.844	0.0033
2018	diaphragmatic vs accessory	0.039	-0.015 to 0.093	0.2495
2019	apical vs accessory	0.077	-0.070 to 0.224	0.5345
2019	apical vs cardiac	-0.175	-0.495 to 0.145	0.4984
2019	apical vs diaphragmatic	0.089	-0.090 to 0.268	0.5798
2019	cardiac vs accessory	0.252	-0.213 to 0.716	0.5039
2019	cardiac vs diaphragmatic	0.263	-0.233 to 0.760	0.5233
2019	diaphragmatic vs accessory	-0.012	-0.051 to 0.028	0.8728
2020	apical vs accessory	0.036	-0.053 to 0.125	0.7244
2020	apical vs cardiac	-0.144	-0.464 to 0.176	0.6555
2020	apical vs diaphragmatic	0.038	-0.054 to 0.130	0.7141
2020	cardiac vs accessory	0.180	-0.228 to 0.588	0.6680
2020	cardiac vs diaphragmatic	0.182	-0.229 to 0.593	0.6664
2020	diaphragmatic vs accessory	-0.002	-0.015 to 0.011	0.9849
2021	apical vs accessory	0.000	-0.016 to 0.016	1.0000
2021	apical vs cardiac	-0.072	-0.247 to 0.103	0.7164
2021	apical vs diaphragmatic	0.018	-0.025 to 0.061	0.7154
2021	cardiac vs accessory	0.072	-0.099 to 0.243	0.7026
2021	cardiac vs diaphragmatic	0.090	-0.127 to 0.306	0.7118
2021	diaphragmatic vs accessory	-0.018	-0.066 to 0.030	0.7774
2022	apical vs accessory	0.011	-0.019 to 0.040	0.7874
2022	apical vs cardiac	-0.038	-0.134 to 0.058	0.7364
2022	apical vs diaphragmatic	0.016	-0.026 to 0.059	0.7584
2022	cardiac vs accessory	0.049	-0.073 to 0.171	0.7321
2022	cardiac vs diaphragmatic	0.054	-0.082 to 0.191	0.7353
2022	diaphragmatic vs accessory	-0.006	-0.025 to 0.014	0.8799

Table S35: Estimated probabilities of structural zeros for the anatomical lobe region model by lobe region and year

Year	Lobe type	Probability	95% CI
2018	accessory	0.970	0.943 to 0.984
2018	apical	0.354	0.227 to 0.506
2018	cardiac	0.329	0.210 to 0.475
2018	diaphragmatic	0.707	0.549 to 0.826
2019	accessory	0.921	0.868 to 0.954
2019	apical	0.822	0.725 to 0.890
2019	cardiac	0.751	0.634 to 0.840
2019	diaphragmatic	0.901	0.838 to 0.942
2020	accessory	0.953	0.916 to 0.974
2020	apical	0.933	0.887 to 0.961
2020	cardiac	0.866	0.789 to 0.918
2020	diaphragmatic	0.967	0.944 to 0.981

Year	Lobe type	Probability	95% CI
2021	accessory	0.968	0.944 to 0.982
2021	apical	0.938	0.895 to 0.964
2021	cardiac	0.921	0.870 to 0.953
2021	diaphragmatic	0.966	0.941 to 0.981
2022	accessory	0.982	0.966 to 0.990
2022	apical	0.963	0.935 to 0.979
2022	cardiac	0.946	0.908 to 0.969
2022	diaphragmatic	0.972	0.949 to 0.985

Table S36: Pairwise comparisons of the odds of structural zeros among anatomical lobe regions within each year. Note: Odds ratios and Tukey-adjusted *p*-values are shown

Year	Contrast	Odds ratio	95% CI	Adjusted *p*-value
2018	apical / accessory	0.017	0.009 to 0.030	<0.0001
2018	apical / cardiac	1.119	0.789 to 1.587	0.8416
2018	apical / diaphragmatic	0.227	0.132 to 0.391	<0.0001
2018	cardiac / accessory	0.015	0.009 to 0.026	<0.0001
2018	cardiac / diaphragmatic	0.203	0.121 to 0.342	<0.0001
2018	diaphragmatic / accessory	0.074	0.038 to 0.146	<0.0001
2019	apical / accessory	0.395	0.295 to 0.530	<0.0001
2019	apical / cardiac	1.530	1.252 to 1.869	<0.0001
2019	apical / diaphragmatic	0.505	0.392 to 0.651	<0.0001
2019	cardiac / accessory	0.258	0.197 to 0.338	<0.0001
2019	cardiac / diaphragmatic	0.330	0.262 to 0.417	<0.0001
2019	diaphragmatic / accessory	0.783	0.573 to 1.069	0.1804
2020	apical / accessory	0.683	0.434 to 1.076	0.1354
2020	apical / cardiac	2.139	1.680 to 2.722	<0.0001
2020	apical / diaphragmatic	0.467	0.340 to 0.641	<0.0001
2020	cardiac / accessory	0.319	0.207 to 0.492	<0.0001
2020	cardiac / diaphragmatic	0.218	0.164 to 0.289	<0.0001
2020	diaphragmatic / accessory	1.464	0.912 to 2.349	0.1637
2021	apical / accessory	0.501	0.351 to 0.714	<0.0001
2021	apical / cardiac	1.289	0.981 to 1.692	0.0786
2021	apical / diaphragmatic	0.527	0.377 to 0.737	<0.0001
2021	cardiac / accessory	0.389	0.288 to 0.525	<0.0001
2021	cardiac / diaphragmatic	0.409	0.309 to 0.542	<0.0001
2021	diaphragmatic / accessory	0.950	0.664 to 1.359	0.9825
2022	apical / accessory	0.483	0.283 to 0.822	0.0025
2022	apical / cardiac	1.481	1.029 to 2.132	0.0289
2022	apical / diaphragmatic	0.737	0.444 to 1.222	0.4076
2022	cardiac / accessory	0.326	0.200 to 0.530	<0.0001
2022	cardiac / diaphragmatic	0.498	0.315 to 0.786	0.0005
2022	diaphragmatic / accessory	0.655	0.360 to 1.191	0.2642

Table S37: Model specification for the binary sensitivity analysis of lesion presence by anatomical lobe region

**Item**	**Specification**
Response	Lobe-level lesion presence (binary: present versus absent)
Model	Generalized linear mixed-effects model
Distribution	Binomial
Link	Logit
Fixed effects	Lobe region × year + farm size
Random effects	Random intercepts for farm and animal-ID nested within farm
Predictions	Lobe region probabilities within year, with random-effects set to zero and averaged over farm size categories

× indicates interaction between the two variables

Table S38: Estimated probability of non-zero Madec score by anatomical lobe region within each year from the binomial mixed-effects sensitivity model. Note: Probabilities and 95% confidence intervals are back-transformed from the logit scale and are averaged over farm size categories

Year	Lobe region	Probability	SE	95% CI
2018	accessory	0.015	0.005	0.008 to 0.027
2018	apical	0.227	0.047	0.148 to 0.333
2018	cardiac	0.309	0.057	0.209 to 0.431
2018	diaphragmatic	0.047	0.012	0.028 to 0.079
2019	accessory	0.049	0.013	0.029 to 0.081
2019	apical	0.087	0.021	0.053 to 0.139
2019	cardiac	0.161	0.036	0.102 to 0.245
2019	diaphragmatic	0.047	0.012	0.028 to 0.077
2020	accessory	0.023	0.006	0.013 to 0.040
2020	apical	0.041	0.011	0.025 to 0.068
2020	cardiac	0.100	0.024	0.062 to 0.157
2020	diaphragmatic	0.021	0.006	0.012 to 0.035
2021	accessory	0.026	0.007	0.015 to 0.044
2021	apical	0.032	0.008	0.019 to 0.053
2021	cardiac	0.061	0.015	0.037 to 0.099
2021	diaphragmatic	0.021	0.006	0.012 to 0.035
2022	accessory	0.013	0.004	0.007 to 0.024
2022	apical	0.022	0.006	0.013 to 0.037
2022	cardiac	0.038	0.010	0.023 to 0.064
2022	diaphragmatic	0.013	0.004	0.008 to 0.023

Table S39: Pairwise comparisons of lesion presence among anatomical lobe regions within each year from the binomial mixed-effects sensitivity model. Note: Odds ratios and Tukey-adjusted *p*-values are shown

Year	Contrast	Odds ratio	95% CI	Adjusted *p*-value
2018	apical / accessory	19.628	12.567 to 30.658	<0.0001
2018	apical / cardiac	0.657	0.557 to 0.775	<0.0001
2018	apical / diaphragmatic	5.931	4.721 to 7.451	<0.0001
2018	cardiac / accessory	29.877	19.139 to 46.640	<0.0001
2018	cardiac / diaphragmatic	9.028	7.199 to 11.323	<0.0001

Year	Contrast	Odds ratio	95% CI	Adjusted p-value
2018	diaphragmatic / accessory	3.309	2.077 to 5.272	<0.0001
2019	apical / accessory	1.860	1.499 to 2.308	<0.0001
2019	apical / cardiac	0.496	0.428 to 0.574	<0.0001
2019	apical / diaphragmatic	1.950	1.640 to 2.319	<0.0001
2019	cardiac / accessory	3.750	3.041 to 4.625	<0.0001
2019	cardiac / diaphragmatic	3.932	3.331 to 4.640	<0.0001
2019	diaphragmatic / accessory	0.954	0.760 to 1.197	0.9510
2020	apical / accessory	1.796	1.339 to 2.410	<0.0001
2020	apical / cardiac	0.386	0.321 to 0.463	<0.0001
2020	apical / diaphragmatic	2.022	1.593 to 2.568	<0.0001
2020	cardiac / accessory	4.656	3.524 to 6.152	<0.0001
2020	cardiac / diaphragmatic	5.243	4.208 to 6.532	<0.0001
2020	diaphragmatic / accessory	0.888	0.647 to 1.220	0.7720
2021	apical / accessory	1.229	0.937 to 1.612	0.2074
2021	apical / cardiac	0.506	0.418 to 0.612	<0.0001
2021	apical / diaphragmatic	1.562	1.240 to 1.968	<0.0001
2021	cardiac / accessory	2.430	1.880 to 3.140	<0.0001
2021	cardiac / diaphragmatic	3.088	2.496 to 3.821	<0.0001
2021	diaphragmatic / accessory	0.787	0.590 to 1.049	0.1397
2022	apical / accessory	1.626	1.056 to 2.503	0.0200
2022	apical / cardiac	0.555	0.422 to 0.730	<0.0001
2022	apical / diaphragmatic	1.643	1.167 to 2.312	0.0011
2022	cardiac / accessory	2.927	1.940 to 4.415	<0.0001
2022	cardiac / diaphragmatic	2.958	2.157 to 4.056	<0.0001
2022	diaphragmatic / accessory	0.989	0.625 to 1.566	0.9999

Conclusion of the binomial sensitivity analysis: the binomial model broadly supported the patterns observed in the primary analysis for the anatomical lobe region, with the cardiac lobe generally showing the greatest lesion involvement, followed by the apical lobe, whereas the accessory and diaphragmatic lobes showed lower involvement and alternated in their relative ordering across years.

Table S40: Model specifications for the left-versus-right lung analysis

Item	Specification
Response	Lobe-specific lesion grade
Distribution and link	Zero-inflated negative binomial (nbinom2), log link
Conditional component	Lung side + year + farm size
Zero-inflation component	Year × lung side
Random effects in conditional component	Farm; animal ID nested within farm
Random effects in zero-inflation component	Farm; animal ID nested within farm
Model parametrisation	Conditional component: ~ lung side + year + farm size + (1 | farm/animal ID)<br>Zero-inflation component: ~year × lung side + (1 | farm/animal ID)

× indicates interaction between the two variables

Table S41: Comparison of NB2 and NB1 parameterisations for the left-versus-right lung model using information criteria

Distribution	AIC	BIC
Negative binomial 2 (NB2)	59,586.646	59,800.595
Negative binomial 1 (NB1)	59,586.646	59,800.595

Lower AIC and BIC indicate better relative support. NB1 and NB2 are non-nested distributional families; therefore, likelihood-ratio *p*-values are not reported

Table S42: DHARMa simulation-based residual diagnostics comparing NB2 and NB1 parameterisations for the left-versus-right lung model

Distribution	Uniformity D	Uniformity *p*-value	Dispersion ratio	Dispersion *p*-value	Zero ratio observed/simulated	Zero-inflation *p*-value
NB2	0.0264	<0.0001	0.589	0.0680	1.033	0.4180
NB1	0.0266	<0.0001	0.593	0.0560	1.032	0.4180

Uniformity is assessed by a Kolmogorov-Smirnov test; dispersion compares observed residual variability with simulations; the zero-inflation test compares observed and simulated numbers of zeros

Model selection conclusion: AIC and BIC were identical for the NB1 and NB2 parameterisations. Based on the overall residual diagnostic assessment, NB2 was retained for the final left-versus-right lung model.

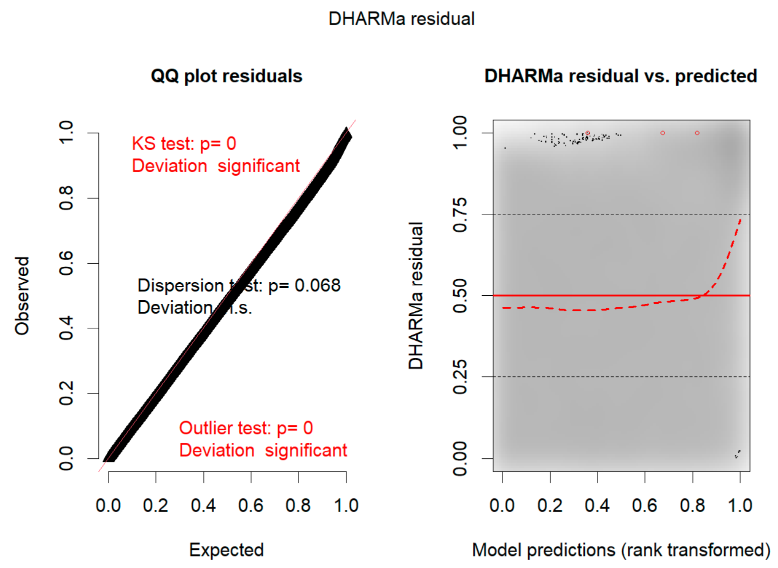

Figure S10: DHARMa residual diagnostics for the left-versus-right lung model. Note: The Q-Q plot compares simulated residuals with their expected uniform distribution, while the residual-versus-predicted plot shows residuals across rank-transformed fitted values. The Kolmogorov-Smirnov and outlier tests indicated significant deviations. Given the large number of observations used in the model (n = 80,994), the tests have high power to detect small deviations

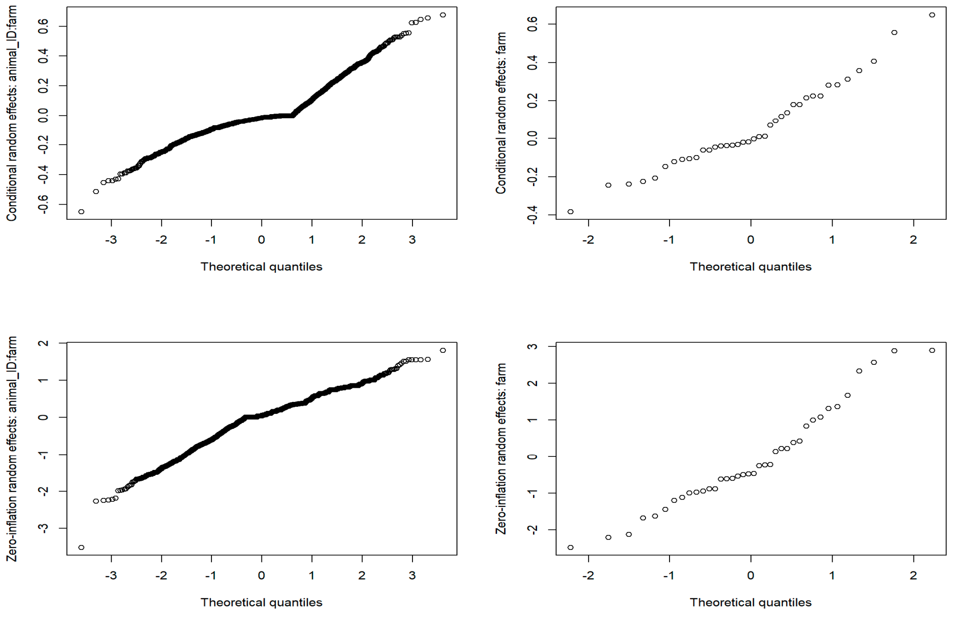

Figure S11: Normal Q-Q plots of random effects from the left-versus-right lung model. Note: Panels show animal-ID (upper-left and bottom-left) and farm (upper-right and bottom-right) random effects for the conditional (upper) and zero-inflation (bottom) components

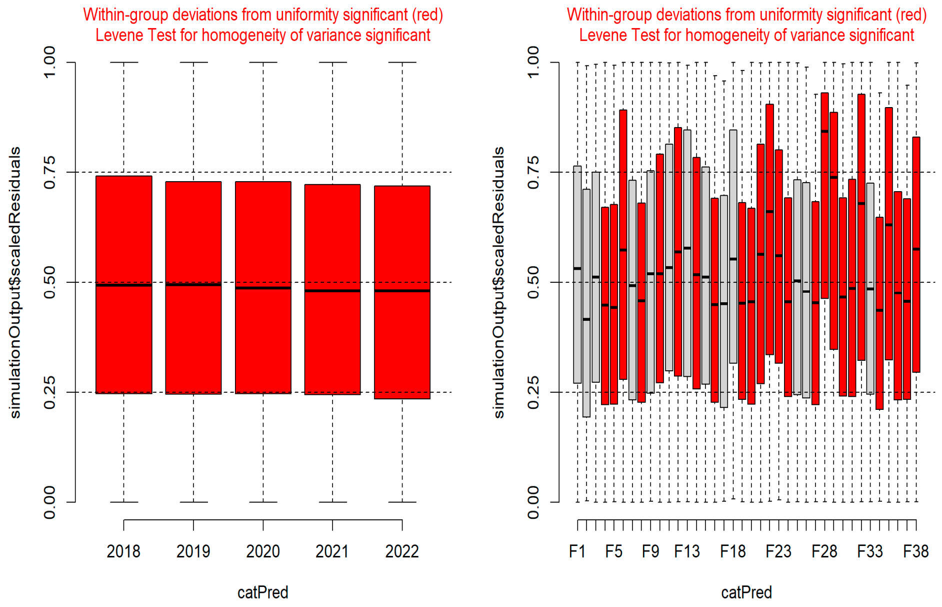

Figure S12: DHARMa residual diagnostics for the left-versus-right lung model, stratified by year (left) and anonymised farm (right). Note: Significant within-group deviations from uniformity were detected. Given the large sample size (n = 80,994), the tests are highly sensitive to relatively small deviations

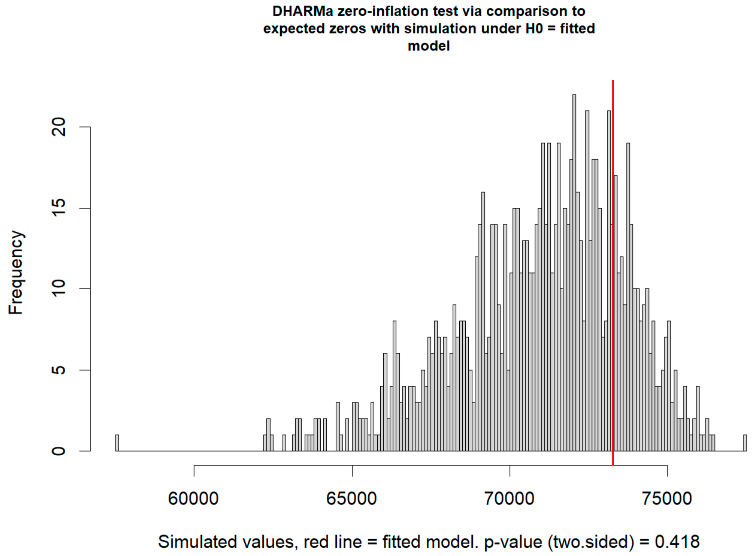

Figure S13: DHARMa zero-inflation diagnostic for the left-versus-right lung model. Note: The histogram shows simulated numbers of zeros under the fitted model; the red line shows the observed number of zeros. The DHARMa zero-inflation test was not significant (p-value = 0.4180), indicating no evidence that the fitted model inadequately accounted for the observed frequency of zeros

Table S43: Omnibus Wald χ^2 tests of fixed effects in the conditional component of the left-versus-right lung model. Note: H0: the conditional mean lobe score does not differ among the levels of each model effect

Effect	Wald χ^2	df	*p*-value
Lung side	8.22	1	0.0041
Year	48.39	4	<0.0001
Farm size	3.15	2	0.2070

Table S44: Fixed-effect estimates for the conditional component of the left-versus-right lung model. Note: Estimates are on the log scale

Predictor	Estimate	SE	95% CI	z	*p*-value
(Intercept)	-0.137	0.125	-0.381 to 0.107	-1.10	0.2724
Lung side right	-0.067	0.023	-0.113 to -0.021	-2.87	0.0041
Year 2019	0.369	0.072	0.227 to 0.510	5.09	<0.0001
Year 2020	0.505	0.106	0.298 to 0.712	4.78	<0.0001
Year 2021	0.289	0.123	0.047 to 0.530	2.34	0.0190
Year 2022	0.247	0.137	-0.020 to 0.515	1.81	0.0700
Farm size medium	-0.044	0.127	-0.292 to 0.204	-0.35	0.7291
Farm size small	0.159	0.122	-0.080 to 0.397	1.30	0.1919

Table S45: Fixed-effect estimates for the zero-inflation component of the left-versus-right lung model. Note: Estimates are on the logit scale

Predictor	Estimate	SE	95% CI	z	*p*-value
(Intercept)	0.591	0.290	0.022 to 1.160	2.04	0.0418
Year 2019	1.249	0.116	1.022 to 1.477	10.75	<0.0001
Year 2020	2.070	0.155	1.766 to 2.375	13.34	<0.0001
Year 2021	2.257	0.165	1.933 to 2.580	13.68	<0.0001
Year 2022	2.634	0.173	2.295 to 2.973	15.24	<0.0001
Lung side right	-0.379	0.080	-0.536 to -0.222	-4.73	<0.0001
Year 2019 × Lung side right	0.017	0.095	-0.169 to 0.202	0.18	0.8608
Year 2020 × Lung side right	0.084	0.100	-0.113 to 0.281	0.84	0.4030
Year 2021 × Lung side right	0.207	0.103	0.006 to 0.409	2.02	0.0437
Year 2022 × Lung side right	0.305	0.124	0.063 to 0.548	2.47	0.0136

× indicates interaction between the two variables

Table S46: Overall model-based predicted mean Madec scores for the left-versus-right lung model within each year. Note: Predicted means combine the conditional and zero-inflation components and are averaged over farm size categories; 95% confidence intervals are simulation-based and were obtained from the joint asymptotic sampling distribution of the conditional and zero-inflation fixed-effect coefficients; random-effects were set to zero

Year	Lung side	Predicted mean	Simulation-based 95% CI
2018	left	0.324	0.223 to 0.448
2018	right	0.380	0.272 to 0.504
2019	left	0.180	0.113 to 0.280
2019	right	0.228	0.147 to 0.344
2020	left	0.098	0.060 to 0.160
2020	right	0.121	0.074 to 0.193

Year	Lung side	Predicted mean	Simulation-based 95% CI
2021	left	0.067	0.040 to 0.109
2021	right	0.073	0.045 to 0.119
2022	left	0.045	0.026 to 0.074
2022	right	0.045	0.027 to 0.074

Table S47: Pairwise comparisons of overall model-based predicted mean Madec scores between the left and right lung sides within each year. Note: Differences are on the overall response scale and are averaged over farm size categories; random-effects were set to zero; pairwise differences, 95% confidence intervals, and *p*-values were obtained using emmeans

Year	Contrast	Difference	95% CI	*p*-value
2018	left vs right	-0.056	-0.088 to -0.024	0.0006
2019	left vs right	-0.048	-0.110 to 0.013	0.1238
2020	left vs right	-0.022	-0.060 to 0.015	0.2431
2021	left vs right	-0.007	-0.020 to 0.007	0.3496
2022	left vs right	-0.000	-0.008 to 0.008	0.9700

Table S48: Estimated probabilities of structural zeros for the left-versus-right lung model by lung side and year

Year	Lung side	Probability	95% CI
2018	left	0.644	0.505 to 0.761
2018	right	0.553	0.409 to 0.689
2019	left	0.863	0.788 to 0.914
2019	right	0.814	0.722 to 0.881
2020	left	0.935	0.895 to 0.960
2020	right	0.914	0.864 to 0.947
2021	left	0.945	0.911 to 0.967
2021	right	0.936	0.896 to 0.961
2022	left	0.962	0.936 to 0.977
2022	right	0.959	0.932 to 0.976

Table S49: Pairwise comparisons of the odds of structural zeros between the left and right lung sides within each year. Note: Odds ratios and Tukey-adjusted *p*-values are shown

Year	Contrast	Odds ratio	95% CI	Adjusted *p*-value
2018	left / right	1.460	1.248 to 1.709	<0.0001
2019	left / right	1.436	1.279 to 1.613	<0.0001
2020	left / right	1.343	1.178 to 1.530	<0.0001
2021	left / right	1.187	1.034 to 1.362	0.0149
2022	left / right	1.076	0.887 to 1.305	0.4567

Table S50: Model specification for the binary sensitivity analysis of lesion presence by lung side

Item	Specification
Response	Lobe-level lesion presence (binary: present versus absent)
Model	Generalized linear mixed-effects model
Distribution	Binomial
Link	Logit
Fixed effects	Lung side × year + farm size
Random effects	Random intercepts for farm and animal-ID nested within farm
Predictions	Left- and right-side probabilities within year, with random effects set to zero and averaged over farm size categories

× indicates interaction between the two variables

Table S51: Estimated probability of non-zero Madec scores for the left and right lung sides within each year from the binomial mixed-effects sensitivity model. Note: Probabilities and 95% confidence intervals are back-transformed from the logit scale and are averaged over farm size categories

Year	Lung side	Probability	SE	95% CI
2018	left	0.181	0.038	0.118 to 0.267
2018	right	0.218	0.043	0.145 to 0.315
2019	left	0.091	0.021	0.057 to 0.141
2019	right	0.117	0.026	0.074 to 0.178
2020	left	0.049	0.012	0.030 to 0.078
2020	right	0.061	0.014	0.038 to 0.096
2021	left	0.037	0.009	0.023 to 0.060
2021	right	0.041	0.010	0.026 to 0.066
2022	left	0.025	0.006	0.015 to 0.041
2022	right	0.026	0.007	0.016 to 0.042

Table S52: Pairwise comparisons of non-zero Madec scores for the left and right lung sides within each year from the binomial mixed-effects sensitivity model. Note: Odds ratios and Tukey-adjusted *p*-values are shown

Year	Contrast	Odds ratio	95% CI	Adjusted *p*-value
2018	left / right	0.792	0.710 to 0.884	<0.0001
2019	left / right	0.758	0.690 to 0.833	<0.0001
2020	left / right	0.790	0.702 to 0.889	<0.0001
2021	left / right	0.891	0.788 to 1.008	0.0678
2022	left / right	0.971	0.811 to 1.163	0.7480

Conclusion of the binomial sensitivity analysis: the binomial model broadly supported the pattern observed in the primary analysis for the left and right lung sides, with greater lesion involvement on the right than on the left side in the earlier years and a progressive reduction in this difference over time.
